# Construction of an immune-related prognostic model and functional analysis of CEBPB in uveal melanoma: A STROBE-compliant observational study

**DOI:** 10.1097/MD.0000000000042574

**Published:** 2025-06-20

**Authors:** Yulin Tao, Yirui Peng, Haibo Zhu, Minqi Xiong, Qiong Zhou, Jun Ouyang

**Affiliations:** a Department of Ophthalmology, The Second Affiliated Hospital of Anhui Medical University, Hefei, Anhui Province, P. R. China; b Department of Ophthalmology, Jiujiang No. 1 People’s Hospital, Jiujiang, Jiangxi Province, P. R. China; c Department of Ophthalmology, The First Affiliated Hospital, Jiangxi Medical College, Nanchang University, Nanchang, Jiangxi Province, P. R. China; d School of Life Sciences, Xiamen University, Xiamen, Fujian Province, P. R. China; e Department of Biomedical Informatics and Data Science, School of Medicine, Johns Hopkins University, Baltimore, MD.

**Keywords:** Bioinformatics, CEBPB, Immune-related genes, Prognostic prediction model, Uveal melanoma

## Abstract

Uveal melanoma (UM) is a primary intraocular malignancy with a high-risk of metastasis. Currently, there are no studies that construct prognostic models based on immune-related molecular subtypes. We performed unsupervised clustering of immune cell infiltration matrices based on the the cancer genome atlas-uveal melanoma (TCGA-UVM) dataset, identifying 2 clusters with distinct expression patterns of immune checkpoint and immune activation related genes. gene ontology/Kyoto Encyclopedia of Genes and Genomes enrichment analysis revealed that genes in the immune-related gene modules identified by WGCNA were associated with immune activity and cell proliferation. Using Cox and LASSO regression analysis based on the immune-related gene modules to construct a prognostic model. The prognostic model was validated in external datasets of Gene Expression Omnibus (GEO) database. We constructed a prognostic model comprising genes S100A4, KCNIP3, PARP8, ORAI2, MMP12, ISG20, MMP9, and CEBPB. The model stratified patients into high and low-risk groups, with the high-risk group showing poorer prognosis. The model’s predictive accuracy was validated with the AUC values exceeding 0.8 for 1-year, 3-year, and 5-year survival rates and confirmed in external datasets GSE22138 and GSE84976. Differential gene analysis between risk groups highlighted the association with immune response and cell proliferation functions. The CEBPB gene in the model played crucial roles in tumor progression. In vivo and in vitro experiments validated the impact of CEBPB on the biological functions of UM. Experiments in UM cells revealed that CEBPB promoted cell proliferation, migration and invasion, as well as suppressing apoptosis, indicating its potential as a therapeutic target. The prognostic model based on 8 immune-related genes effectively predicted the survival outcomes of UM. Knockdown of CEBPB significantly reduced the progression of UM, suggesting that it could be a potential therapeutic target for UM.

## 1. Introduction

Uveal melanoma (UM) is the most common primary intraocular malignancy in adults.^[1]^ Despite its rarity, with an incidence of approximately 5.1 per million individuals per year, UM poses significant management challenges due to its high propensity for metastasis and associated mortality.^[2,3]^ The current standard treatments for UM, primarily consisting of surgery and radiotherapy, are effective for localized tumors.^[1]^ There is a pressing need for novel therapeutic strategies, particularly for metastatic UM.

Prognostic models play a pivotal role in cancer care, guiding treatment decisions and aiding in the prediction of patient outcomes. In UM, existing models rely on clinicopathological and genetic factors.^[4]^ Previously, there was no analysis of molecular subtypes based on immune infiltration characteristics for UM. This gap underscores the importance of integrating novel biomarkers that reflect the tumor’s immunogenicity into prognostic frameworks. Recent advances had identified several biomarkers associated with immunotherapeutic responses in other malignancies.^[5]^ These contained the expression of immune checkpoint molecules, tumor-infiltrating lymphocyte patterns, and cytokine profiles.^[6]^ Our study aimed to construct a prognostic model that incorporated immune-related genes based on the cancer genome atlas-uveal melanoma (TCGA-UVM) dataset. The model was validated in GEO profile of UM patients, and evaluated its efficacy in predicting patient outcomes. We also attempted to validate the genes in the model at the cellular level.

## 2. Materials and methods

### 2.1. Data preprocessing

Expression profile data about UM were retrieved from TCGA database, encompassing a cohort of 80 cancer specimens. Additionally, transcriptomic sequencing data for UM tissues were procured from the Gene Expression Omnibus (GEO) database, and the accession numbers are GSE22138 and GSE84976. The GSE22138 dataset included 63 cancer specimens, each accompanied by survival data. Similarly, the GSE84976 dataset contained 28 cancer specimens, all with available survival information. These oncological samples, documented with survival metrics, were employed for the prognostic model validation. For the transcriptomic data mentioned above, we integrated the samples and employed canonical correlation analysis to eliminate batch effects, thereby minimizing inconsistencies arising from batch variability.

### 2.2. Immune infiltration analysis

We calculated the infiltration levels of 22 types of immune cells in all UM samples from TCGA. For the CIBERSORT analysis, utilizing the “deconvo_tme” function from the R package IOBR,^[7]^ which was predicated upon the LM22 signature gene set, we quantified the infiltration scores for 22 immune cell types within each specimen to reflect their respective levels of tumor microenvironment (TME) infiltration, setting the “perm” parameter to 200. Subsequently, an unsupervised clustering analysis was conducted on the immune cell infiltration matrix for all UM samples. We applied the “ConsensusClusterPlus” function from the R package ConsensusClusterPlus to ascertain the optimal number of clusters.^[8]^ This function facilitated unsupervised clustering using the k-means algorithm, with parameters set as follows: maxK = 6, reps = 1000, pItem = 0.8, clusterAlg = “km,” innerLinkage = “ward.D2,” finalLinkage = “ward.D2,” and distance = “euclidean.” The clustering outcomes were visualized using principal component analysis (PCA).

### 2.3. Analysis of immune characteristics between different molecular subtypes

Composite violin and box plots were employed to examine the expression patterns of PD-1 (PDCD1) and PDL1 (CD274) across various molecular subtypes. Drawing upon extant literature,^[9]^ genes CTLA4, HAVCR2, IDO1, and LAG3 were delineated as immune checkpoint correlated genes, while CD8A, CXCL10, CXCL9, GZMA, GZMB, IFNG, PRF1, TBX2, and TNF were characterized as markers associated with immune activation. Consequently, we scrutinized the expression profiles of these 2 gene categories within the different subtypes, applying the Wilcoxon rank-sum test for statistical significance assessment.

### 2.4. Weighted gene co-expression network analysis (WGCNA)

R package WGCNA was applied to unearth gene modules associated with subtype grouping labels from differentially expressed genes (DEGs). The function “pickSoftThreshold” was utilized to determine the optimal soft-thresholding power, which subsequently informed the construction of the network via the “adjacency” function. Gene modules were visualized using “plotDendroAndColors,” and the intermodular connectivity was assessed through a correlation heatmap. To identify gene modules associated with the subtype labels, a module-trait relationship analysis was performed, with result visualized by the “labeledHeatmap” function. In pursuit of deciphering the biological functions of the associated gene modules, enrichment analyses were executed employing gene ontology (GO) and Kyoto Encyclopedia of Genes and Genomes (KEGG) methodologies. The R package “clusterProfiler”^[10]^ facilitated the annotation of genes within the associated modules for GO functions and KEGG pathway enrichment, identifying significantly enriched biological attributes, with an adjusted *P*-value threshold set at < 0.05 for the enrichment significance.

### 2.5. Construction and validation of the prognostic prediction model

To elucidate the nexus between the immunological attributes of UM and patient outcomes, a prognostic model was developed based on TCGA-UVM dataset, predicated upon the gene modules identified previously, employing univariate Cox and LASSO regression analyses. The validity and predictive acumen of this model were corroborated using 2 independent external GEO datasets.

Univariate Cox regression analysis was performed with the R package “survival.” Subsequently, the genes that met the statistical threshold were used as input to establish the LASSO regression model using the R package “glmnet.” The “cv.glmnet” function was employed to construct the model, with the “family” parameter set as “cox” and the “alpha” parameter set as 1. Finally, ineffective genes with coefficients of 0 were eliminated from the model. The model was then applied to score the UM dataset from TCGA, and based on the risk scores, all samples were stratified into high- and low-risk groups. Perform stratified survival analysis on high-risk and low-risk groups using the “survival” package in R, and plot the Kaplan–Meier (KM) survival curves to assess the predictive performance of the model.

To validate the effectiveness of the model, the R package “timeROC”^[11]^ was utilized to plot the predicted receiver operating characteristic (ROC) curves of the model for 1-year, 3-year, and 5-year survival rates on the original dataset. Subsequently, in order to assess the applicability of the model, 2 external datasets, GSE22138 and GSE84976, were employed. The same procedure was followed to score the samples based on the risk score, and KM survival curves were plotted for each dataset to evaluate the predictive performance. Univariate Cox regression analysis was performed to evaluate the hazard ratios of prognostic genes on the survival rate of UM disease, and a forest plot was generated.

### 2.6. Assessment of the prognostic risk model

Based on the TCGA-UVM dataset, we utilized univariate and multivariate Cox regression analyses to evaluate the predictive capability of risk scores combined with the clinicopathological characteristics of UM patients for survival probability. The analysis results were presented in forest plots. For the clinical information of the samples in this study, we employed the R package “rms” to construct nomogram to investigate the relationship between clinical factors and prognosis, and plotted calibration curves for 1-year, 2-year, and 3-year survival predictions, respectively.

### 2.7. Differential analysis and biological functional analysis between high-risk and low-risk groups

To further investigate the biological differences between high-risk and low-risk groups, perform differential expression analysis between the groups using the “limma” package in R. Set the statistical significance threshold at adj.*P*-value < 0.05 and |log2FC| > 1. Visualize the significantly DEGs using volcano plots and expression heatmaps based on the “ggplot2”^[12]^ and “pheatmap” packages^[13]^ in R, respectively.

Perform GO and KEGG enrichment analysis on the significantly DEGs identified above using the “clusterProfiler” package in R.^[10]^ Set the statistical significance threshold at adj.*P*-value < .05. Visualize the enrichment analysis results using bar plots and bubble plots.

### 2.8. Conduct single-sample gene set enrichment analysis (ssGSEA)

SsGSEA is a method for gene set enrichment analysis that can be used to analyze the enrichment of pathways or biological processes (BP) in the gene expression data of individual samples. The basic idea behind ssGSEA is to rank the expression values of all genes in a gene set from high to low and then calculate the cumulative distribution function of the gene set on this ranked list. A higher value of the cumulative distribution function indicates that the genes in the gene set are expressed more highly in the sample, reflecting the enrichment level of the gene set in that sample. We used an immune gene set and, for each sample, calculated the ssGSEA score by comparing the gene expression data in that sample with the gene set. These enrichment scores could be used to compare immune differences between different samples.

### 2.9. Immune evaluation (ESTIMATE) analysis

To better understand the impact of genes involved in immunity and stromal cells on prognosis, the ESTIMATE package utilizes the unique properties of cancer sample transcriptomes to infer the content of tumor cells and different infiltrating normal cells. We evaluated the immune activity of samples using the expression profile matrix data from TCGA-UVM dataset through the “ESTIMATE” package in R. The ESTIMATE algorithm was used to calculate the ESTIMATE score, Immune Score, and Stromal Score based on the expression matrix features, quantifying the immune and stromal components of the samples.

### 2.10. Statistical analysis

Statistical analyses were performed using R software (Version 4.1.2). Results were presented as mean ± SD, and all experiments were conducted in triplicate. Statistical significance was defined as *P*-value < 0.05. ns: no significance, *P < .05, **P < .01, ***P < .001, **** *P* < .0001.

For other research methods, such as TIDE analysis, cell culture and transfection, RNA isolation and quantitative real-time PCR (qRT-PCR), cell viability and proliferation assay, cell migration and invasion assay, immunohistochemistry, flow cytometry, please refer to Supplementary Materials 1 to 7, Supplemental Digital Content, https://links.lww.com/MD/P31. The detailed sequences of siRNAs, primer sequences, and clinical information for patients with UM used in this study can be found in Tables S1 to S3, Supplemental Digital Content, https://links.lww.com/MD/P30.

## 3. Results

### 3.1. Clustering of UM samples based on immune characteristics

A schematic representation of the study workflow showed in Figure S1, Supplemental Digital Content, https://links.lww.com/MD/P27.

**Figure 1.**
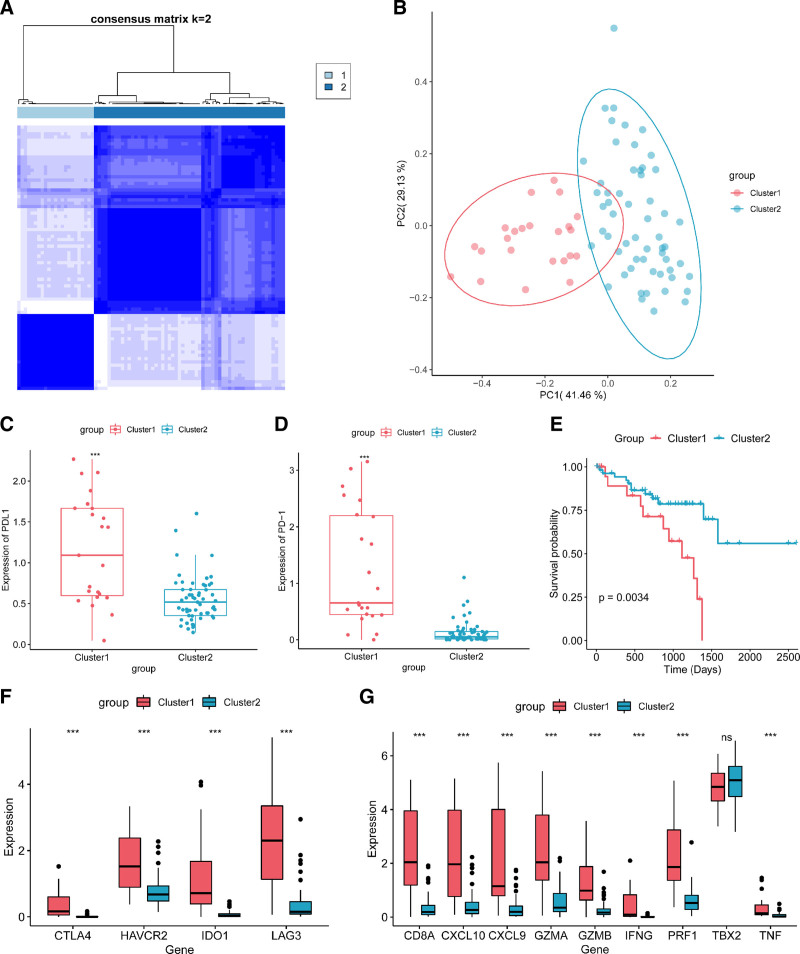
Clustering of tissue samples and analysis of DEGs between subtype groups. (A) Heatmap of consistent clustering (*k* = 2). (B) Dimensionality reduction diagram of k-means clustering results. (C and D) Comparison chart of PD-L1 and PD-1 gene groups. PD-L1 and PD-1 were both highly expressed in cluster1. (E) The KM survival curve result of different clusters in the TCGA-UVM dataset. We observed a significantly lower survival rate in patients from cluster1 compared to cluster2. (F and G) Box plot of immune checkpoint-related and immune activity-related marker genes expression level. Except for TBX2, the expression of other relevant genes was significantly upregulated in cluster1. *P < .05; **P < .01; ***P < .001; ns, *P* > .05. DEGs = differentially expressed genes, KM = Kaplan–Meier, TCGA-UVM = the cancer genome atlas-uveal melanoma.

In this study, focusing on the immune characteristics of UM, an unsupervised clustering analysis was performed on samples from TCGA-UVM dataset. The optimal number of clusters was determined to be 2 through computational evaluation (Fig. 1A). Consequently, using the k-means unsupervised clustering method, all samples were divided into 2 clusters. The results of PCA demonstrated clear and distinct boundaries between the 2 groups of samples in the reduced-dimensional space (Fig. 1B), indicating a well-performed clustering effect.

### 3.2. Analysis of biological characteristics between subtype groups

We examined the expression of PD-1 and PD-L1 in the 2 clusters. Box plots revealed that they were highly expressed in Cluster1 (Fig. 1C and D). Patients in Cluster2 had better survival state (Fig. 1E). To further elucidate the differences in immune features between the 2 sample clusters, box plots were employed to display the expression levels of these genes. The results revealed highly significant differential expression (*P* < .001) of immune checkpoint-related marker genes (Fig. 1F) and immune activity-related marker genes, excluding TBX2 (Fig. 1G), between the 2 clusters. Interestingly, these genes consistently exhibited high expression in Cluster1, which was in line with the previous conclusions of this study.

### 3.3. Identification of immune-related gene modules by WGCNA

**Figure 2.**
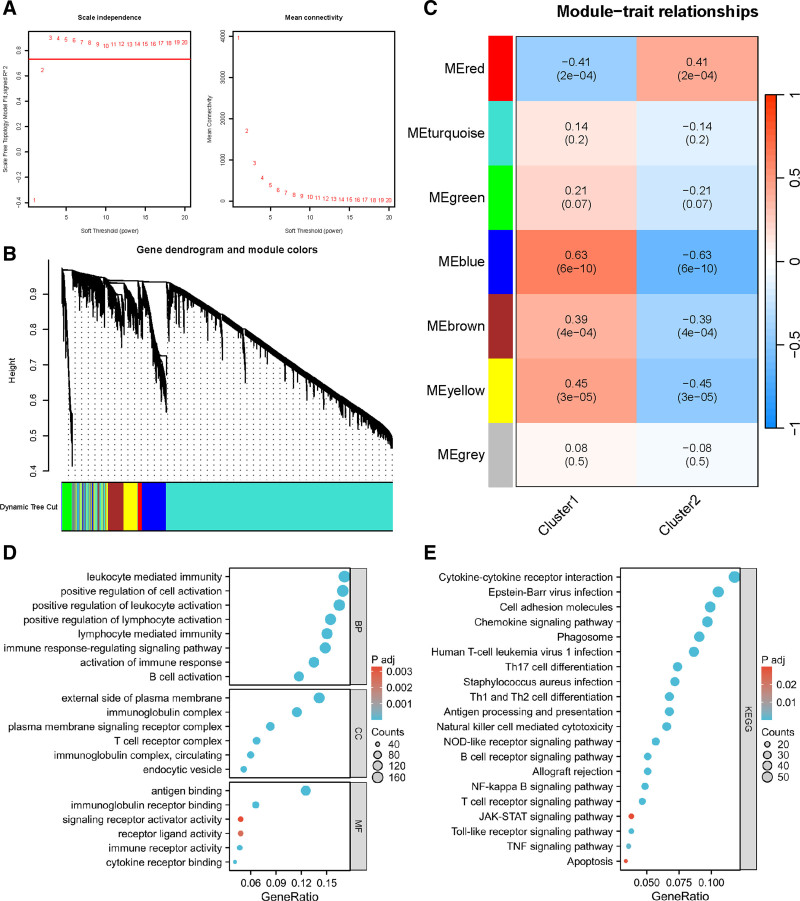
Gene module analysis. (A) Soft threshold selection. (B) WGCNA hierarchical clustering. (C) WGCNA phenotype correlation heat map. The genes in the blue module exhibited the highest correlation with cluster1. (D) Bubble graph of GO enrichment results, including BP, CC, and MF, which were all associated with immunity. (E) Bubble plot of KEGG enrichment results. The results demonstrated that pathways related to both immunity and proliferation were enriched. BP = biological processes, CC = cellular components, GO = gene ontology, KEGG = Kyoto Encyclopedia of Genes and Genomes, MF = molecular functions, WGCNA = weighted gene co-expression network analysis.

Based on the analysis, cluster1 exhibited complex and active immune features. To further explore gene modules and identify immune-related gene modules associated with cluster1, this study employed WGCNA. The optimal soft threshold for WGCNA was determined to be 3 (Fig. 2A). Gene modules were identified by a stepwise network construction approach (Fig. 2B). The result revealed the identification of 7 gene modules, and correlation analysis of the 7 gene modules (Fig. 2C) demonstrated the strongest correlation between the blue module including a total of 1018 genes and cluster1. Therefore, the blue module might represent a key gene module associated with immune features.

To elucidate the biological functions impacted by the 1018 genes within this gene module, GO and KEGG enrichment analyses were performed. The results of the GO enrichment analysis were predominantly related to immune processes (Fig. 2D). The KEGG enrichment analysis results (Fig. 2E) indicated that the top 20 significantly enriched pathways were mainly associated with immune cell activities, which was consistent with the GO enrichment results, thereby validating the conclusions of this study. Additionally, KEGG analysis also enriched pathways related to cell proliferation and apoptosis, highlighting the differences in tumor biological characteristics between the 2 subtype groups.

### 3.4. Construction and validation of the prognostic prediction model

**Figure 3.**
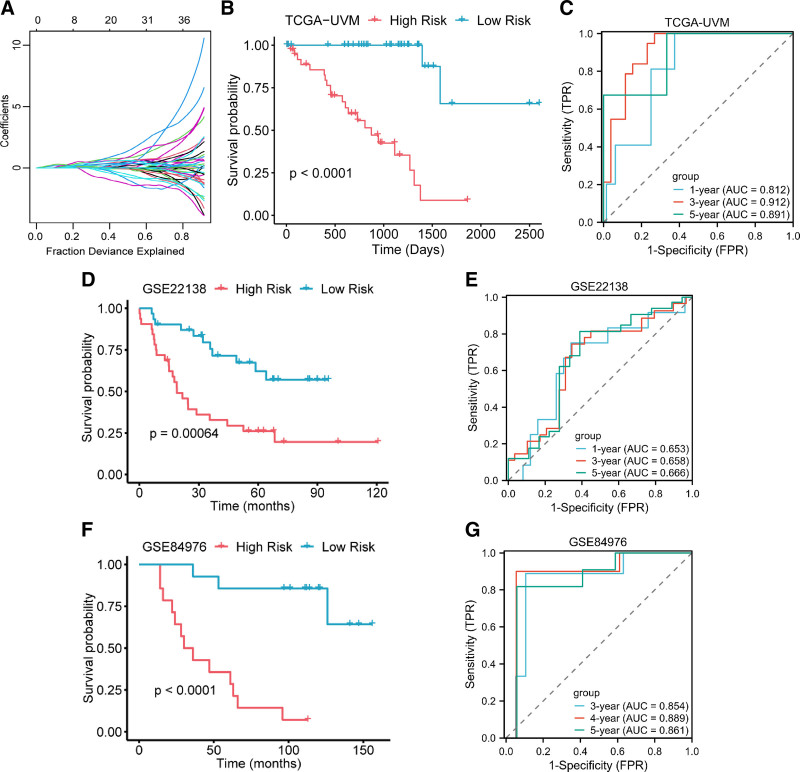
Prognosis prediction model and validation. (A) LASSO regression curve. (B) Survival curves of high and low-risk groups in TCGA-UVM dataset. The survival rate of patients in the high-risk group was significantly lower than that of the low-risk group. (C) TCGA-UVM dataset 1-, 3-, and 5-yr survival prediction ROC curves. (D) GSE22138 dataset high and low-risk group survival curve. (E) GSE22138 dataset 1-, 3-, and 5-yr survival prediction ROC curves. (F) GSE84976 dataset survival curve. (G) GSE84976 dataset 3-, 4-, and 5-yr survival prediction ROC curves. LASSO = least absolute shrinkage and selection operator, ROC = receiver operating characteristic curves, TCGA-UVM = the cancer genome atlas-uveal melanoma.


To elucidate the relationship between immune characteristics and patient prognosis and to apply it to clinical practice, a prognostic prediction model was established by screening the genes within the previously described gene module blue. Based on the survival data from TCGA-UVM samples, univariate Cox regression analysis was conducted to identify genes significantly associated with prognosis. After statistical significance screening, 511 genes remained. A model was constructed using the LASSO regression analysis method based on these 511 genes (Fig. 3A). The results revealed that 8 genes (S100A4, KCNIP3, PARP8, ORAI2, MMP12, ISG20, MMP9, CEBPB) had non-zero coefficients in the regression model and were retained. The final model was presented below:

$$\begin{array}{l} \begin{array}{l} Score\\ =0.0406\times \;Exp_{S100A4}+0.2800\times \;Exp_{KCNIP3}+0.1620\times \;Exp_{PARP8}\;\\ +0.3880\;\times \;Exp_{ORAI2}+0.0830\;\times \;Exp_{MMP12}+0.1860\;\times \;Exp_{ISG20}\;\\ +0.1440\;\times \;Exp_{MMP9}+0.1860\;\times \;Exp_{CEBPB}\; \end{array} \end{array}$$

Based on the above model, all samples were divided into high-risk and low-risk groups. KM survival curves (Fig. 3B) demonstrated a significant difference in prognosis between the 2 groups (*P* < .0001). The area under the ROC curves (AUCs) for the model’s prediction of 1-year, 3-year, and 5-year survival rates were 0.812, 0.912, and 0.891, respectively (Fig. 3C), indicating that the model exhibited good predictive performance. After plotting the ROC curves for survival prediction for each of the 8 genes in the model (Fig. S2, Supplemental Digital Content, https://links.lww.com/MD/P27), we found that the model constructed based on these 8 genes outperformed the predictive performance of each gene individually.

To further validate the gen ceralizability and robustness of the model, 2 external datasets, GSE22138 and GSE84976, were utilized. The results demonstrated significant differences in survival time between the high and low-risk groups in both the GSE22138 (Fig. 3D) and the GSE84976 datasets (Fig. 3F) (*P* < .001). In the GSE22138 dataset, the AUCs for 1-year, 3-year, and 5-year survival rates were 0.653, 0.658, and 0.666, respectively (Fig. 3E). Time-dependent ROC curves were plotted for 3-year, 4-year, and 5-year survival rates in GSE84976 dataset, with AUCs of 0.854, 0.889, and 0.861, respectively (Fig. 3G). The results provided strong evidence for the model’s excellent generalizability, stability, and robustness.

### 3.5. Construction and estimate of the predictive nomogram

**Figure 4.**
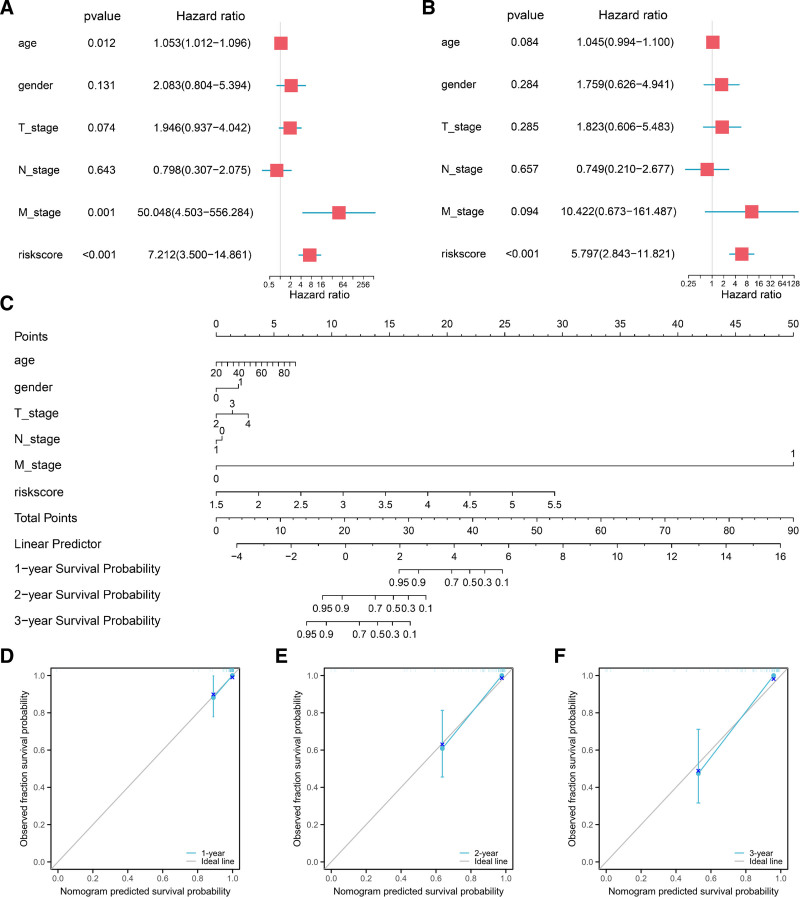
A prognostic nomogram and calibration curves for UM patients were constructed based on the TCGA-UVM dataset. (A) Results from the univariate Cox regression analysis based on risk score and clinical parameters. (B) Results from the multivariate Cox regression analysis based on risk score and clinical parameters. (C) Nomogram for predicting the 1-, 2-, and 3-yr survival probability of UM patients. (D–F) Calibration curves of the nomogram for predicting survival probability at 1, 2, and 3 yr. Cox = proportional hazards model, TCGA-UVM = the cancer genome atlas-uveal melanoma, UM = uveal melanoma.

To investigate the relationship between prognostic characteristics and outcomes in patients with UM, we conducted Cox regression analyses based on TNM stage, gender, age, and risk score. In the univariate Cox regression, age (*P* = .012), M_stage (*P* = .001), and riskScore (*P* < .001) significantly impacted the survival of patients (Fig. 4A). In the multivariate Cox regression, only riskScore showed a significant effect on survival (*P* < .001) (Fig. 4B). Subsequently, a nomogram incorporating common clinical variables and the risk score was developed to quantitatively predict the survival rates of UM patients (Fig. 4C). Calibration curves for 1-year, 2-year, and 3-year survival were plotted (Fig. 4D–F). The calibration curves at different time points demonstrated high predictive accuracy, indicating that our model was highly accurate.

We conducted TIDE (tumor immune dysfunction and exclusion) analysis on the TCGA-UVM dataset. The result was presented in box plots (Fig. S3A, Supplemental Digital Content, https://links.lww.com/MD/P27). We observed that the TIDE scores were significantly lower in the high-risk group compared to the low-risk group (*P* = .005). Based on the high and low-risk score groupings, we compared the differences in ESTIMATEScore, ImmuneScore, and StromalScore among UM samples across different groups, and found that the StromalScore, ImmuneScore, and ESTIMATEScore were all significantly higher in the high-risk group compared to the low-risk group (*P* < .001, Fig. S3B, Supplemental Digital Content, https://links.lww.com/MD/P27). Our findings also revealed that patients in the high StromalScore, ImmuneScore and ESTIMATEScore groups exhibited lower survival rates compared to those in the low groups (Fig. S3C–E, Supplemental Digital Content, https://links.lww.com/MD/P27).

### 3.6. Analysis of biological characteristics between high and low-risk groups

**Figure 5.**
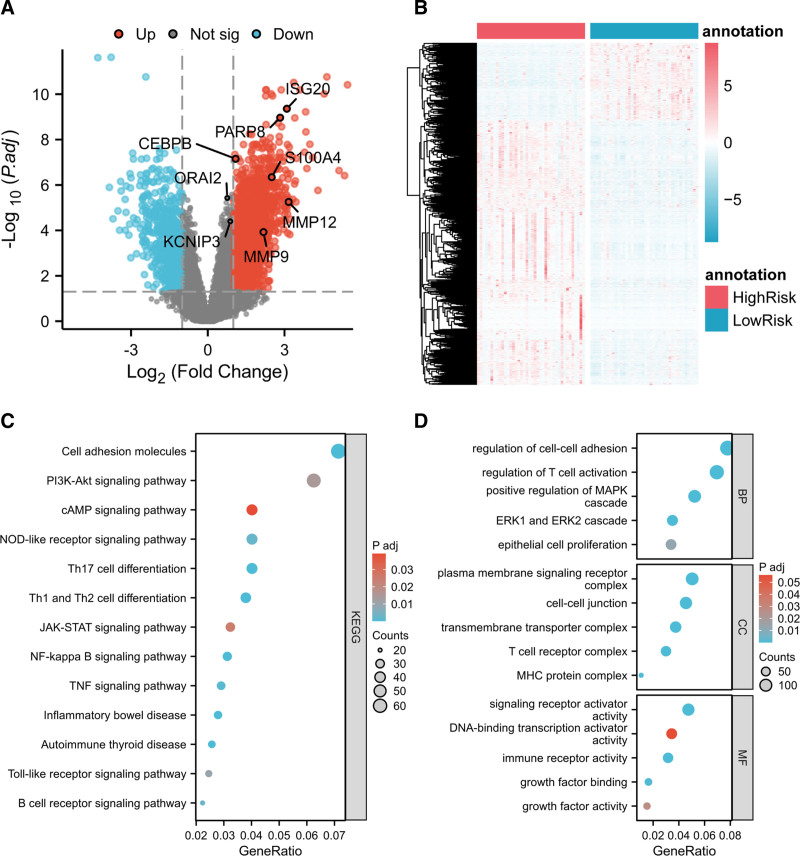
Differential analysis and GO/KEGG enrichment analysis between the high-risk and low-risk groups. (A) The volcano plot of the differential analysis showed that S100A4, KCNIP3, PARP8, ORAI2, MMP12, ISG20, MMP9, and CEBPB genes significantly upregulated in the high-risk group. (B) The heatmap of the differential analysis displayed a trend of upregulation in most genes in the high-risk group. (C) Bubble plot of KEGG enrichment analysis. (D) Bubble plot of GO enrichment analysis. The GO/KEGG analysis revealed that the upregulated genes in the high-risk group were primarily associated with pathways related to immune response and cell proliferation. GO = gene ontology, KEGG = Kyoto Encyclopedia of Genes and Genomes.

We conducted gene differential expression analysis in high and low-risk groups. Following the threshold selection process with the low-risk group as the control group, a total of 3001 significantly DEGs were obtained, including 2320 upregulated genes and 681 downregulated genes (Fig. 5A). The expression heatmap (Fig. 5B) showed that the majority of significantly DEGs exhibited higher expression characteristics in the high-risk group. We conducted GO/KEGG enrichment analysis on the upregulated 2320 genes in the high-risk group. The GO enrichment analysis results for the upregulated genes (Fig. 5D) indicated that in the high-risk group, the BP, cellular components (CC), and molecular functions (MF) were associated with immune-related BP. The KEGG enrichment analysis results (Fig. 5C) revealed that the DEGs in the high-risk group were mainly associated with pathways such as the cAMP signaling pathway, NF-kappa B signaling pathway, PI3K-Akt signaling pathway, etc. These pathways were related to immune response, cell proliferation, and cell growth. Therefore, the high-risk group samples might be characterized by increased malignancy due to higher tumor cell proliferation and enhanced immune infiltration.

### 3.7. Expression of prognostic genes and their impact on survival in patients with UM

**Figure 6.**
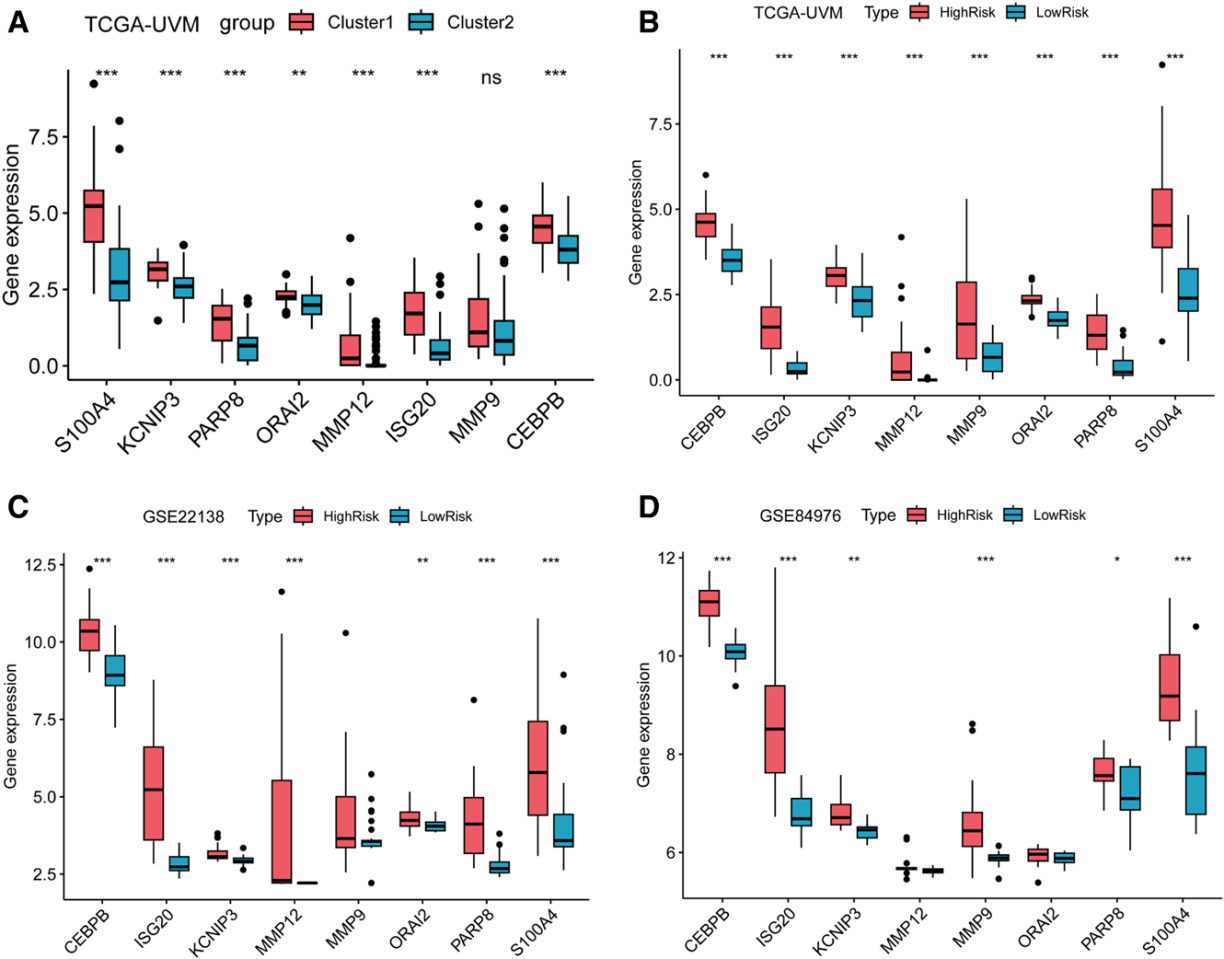
The expression profiles of 8 prognostic genes across different groups. (A) Expression analysis in the TCGA-UVM dataset revealed significantly elevated expression of 7 prognostic genes in cluster1, except for MMP9. (B) All prognostic genes were significantly upregulated in the high-risk group compared to the low-risk group in TCGA-UVM. (C) Except for MMP9, the other 7 prognostic genes were significantly upregulated in the high-risk group compared to the low-risk group in GSE22138 dataset. (D) Except for MMP12 and ORAI2, the other 6 prognostic genes were significantly upregulated in the high-risk group compared to the low-risk group in GSE84976 dataset. TCGA-UVM = the cancer genome atlas-uveal melanoma.

To validate the expression of the 8 prognostic genes in different subgroups, we examined their expression level in cluster1 and cluster2, as well as between the high and low-risk groups in the transcriptome data of TCGA-UVM, GSE22138, and GSE84976. The results revealed that, except for MMP9, the expression of the other genes was significantly elevated in cluster1 compared to cluster2 (Fig. 6A). All genes showed significantly higher expression in the high-risk group of TCGA-UVM compared to the low-risk group (Fig. 6B). Consistent with the expression pattern in cluster1, the genes S100A4, KCNIP3, PARP8, ORAI2, MMP12, ISG20, and CEBPB exhibited significantly higher expression in the high-risk group of the GSE22138 dataset (Fig. 6C). However, in the GSE84976 dataset, only S100A4, KCNIP3, PARP8, ISG20, MMP9, and CEBPB showed significantly higher expression in the high-risk group compared to the low-risk group (Fig. 6D). These results indicated that the expression patterns of the 8 prognostic genes in the high-risk group were well validated in the 2 validation datasets, indirectly affirming the effectiveness of the model.

The survival analysis results indicated that high expression of S100A4, KCNIP3, PARP8, ORAI2, ISG20, and CEBPB genes in the TCGA-UVM transcriptome data significantly decreased the survival rate of UM patients (Fig. S4A–H, Supplemental Digital Content, https://links.lww.com/MD/P27). There was no significant difference in survival probability between patients with high and low expression of the MMP9 gene (Fig. S4I, Supplemental Digital Content, https://links.lww.com/MD/P27). Due to the low expression levels of MMP12, it was not possible to group patients based on its expression values, and therefore corresponding survival analysis results could not be obtained. Univariate Cox regression analysis result indicated that the CEBPB posed the highest risk ratio for mortality in UM patients (Fig. S5, Supplemental Digital Content, https://links.lww.com/MD/P27). Correlation analysis result demonstrated that CEBPB was positively correlated with the other 7 genes, with the highest correlation observed with the PARP8 and ISG20 genes (*R* > 0.75, *P* < .001) (Fig. S6, Supplemental Digital Content, https://links.lww.com/MD/P27).

### 3.8. The impact of CEBPB on immune infiltration in UM patients

**Figure 7.**
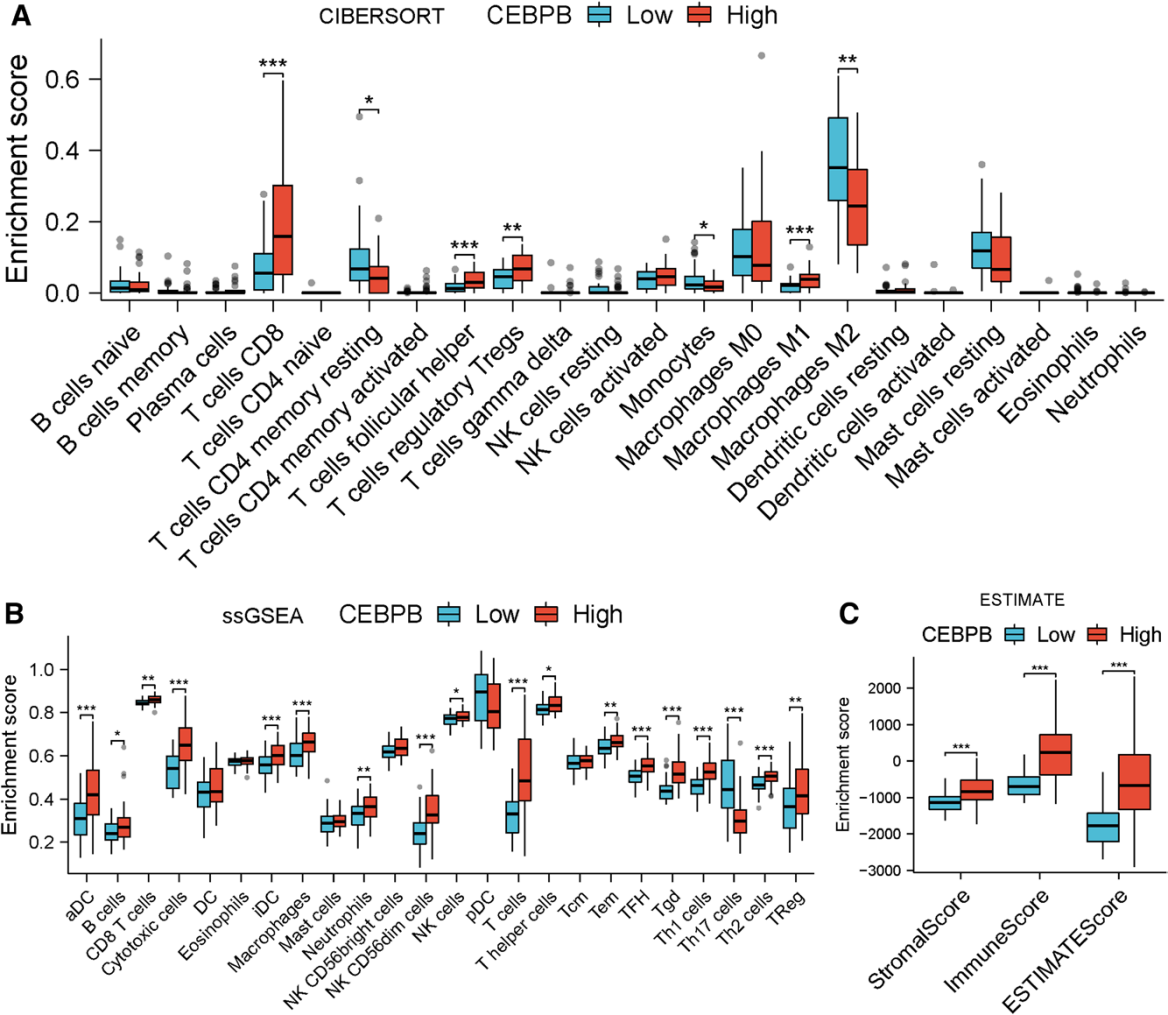
The immune infiltration levels of different cell types and immune scores calculated by 3 algorithms in the high and low CEBPB expression groups of TCGA-UVM patients. (A) The analysis results from the CIBERSORT algorithm indicated a significant increase in the infiltration levels of T cells CD8, T cells follicular helper, T cells regulatory Tregs, and Macrophages M1 in the high CEBPB expression group. (B) The ssGSEA algorithm revealed a significant increase in the infiltration levels of 17 immune cell types in the high CEBPB expression group. (C) The ESTIMATE algorithm demonstrated that the ESTIMATE score, Immune Score, and Stromal Score were significantly higher in the high CEBPB expression group compared to the low-expression group. ssGSEA = single-sample gene set enrichment analysis, TCGA-UVM = the cancer genome atlas-uveal melanoma.

The differences in immune cell infiltration levels between the high and low-expression groups of CEBPB were evaluated using 3 algorithms. The results showed significant differences between the 2 groups in the infiltration levels of T cells CD8, T cells CD4 memory resting, T cells follicular helper, T cells regulatory Tregs, Monocytes, Macrophages M1, and Macrophages M2 as calculated by the CIBERSORT algorithm (Fig. 7A). A correlation heatmap displayed significant correlations of the 8 prognostic genes with 7 immune cells to varying degrees (Fig. S7A, Supplemental Digital Content, https://links.lww.com/MD/P27). Results from the ssGSEA algorithm indicated that the infiltration levels of 18 immune cells were significantly higher in the high-expression group compared to the low-expression group (Fig. 7B, Supplemental Digital Content, https://links.lww.com/MD/P27). A correlation heatmap showed significant correlations of the 8 prognostic genes with 18 immune cells to varying degrees (Fig. S7B, Supplemental Digital Content, https://links.lww.com/MD/P27). The ESTIMATE algorithm calculated that the ESTIMATEScore, ImmuneScore, and StromalScore were significantly higher in the high-expression group compared to the low-expression group (Fig. 7C). These results suggested that the expression level of CEBPB led to significant differences in immune infiltration in tumor tissues of UM patients, indicating that CEBPB might influence the immune microenvironment of UM.

Subsequently, we analyzed the correlation between CEBPB and immune infiltrating cells as well as immune scores (Figs. S8 and S9, Supplemental Digital Content, https://links.lww.com/MD/P27). The results from the CIBERSORT algorithm indicated that CEBPB was significantly positively correlated with Macrophages M1, T cells follicular helper, T cells CD8, and T cells regulatory Tregs (Fig. S9A–D, Supplemental Digital Content, https://links.lww.com/MD/P27), with the highest correlation observed with Macrophages M1 (Fig. S9A, Supplemental Digital Content, https://links.lww.com/MD/P27). Conversely, CEBPB showed a significant negative correlation with Mast cells resting, T cells CD4 memory resting, and Macrophages M2 (Fig. S9E–G, Supplemental Digital Content, https://links.lww.com/MD/P27), with the strongest negative correlation with Macrophages M2 (Fig. S9E, Supplemental Digital Content, https://links.lww.com/MD/P27). The results from the ssGSEA algorithm demonstrated that CEBPB was significantly positively correlated with most immune cells (Fig. S9H–W, Supplemental Digital Content, https://links.lww.com/MD/P27), except for a significant negative correlation with Th17 cells (Fig. S9X, Supplemental Digital Content, https://links.lww.com/MD/P27). This suggested that CEBPB played a critical role in the infiltration of immune cells in UM. Additionally, the ESTIMATE algorithm revealed that CEBPB was positively correlated with ESTIMATEScore, ImmuneScore, and StromalScore, with the strongest correlation observed with ImmuneScore (Fig. S9Y, Supplemental Digital Content, https://links.lww.com/MD/P27). These findings underscored the significant impact of CEBPB on the prognosis of UM patients.

### 3.9. The correlation between CEBPB and immune checkpoint genes as well as immune activation genes

**Figure 8.**
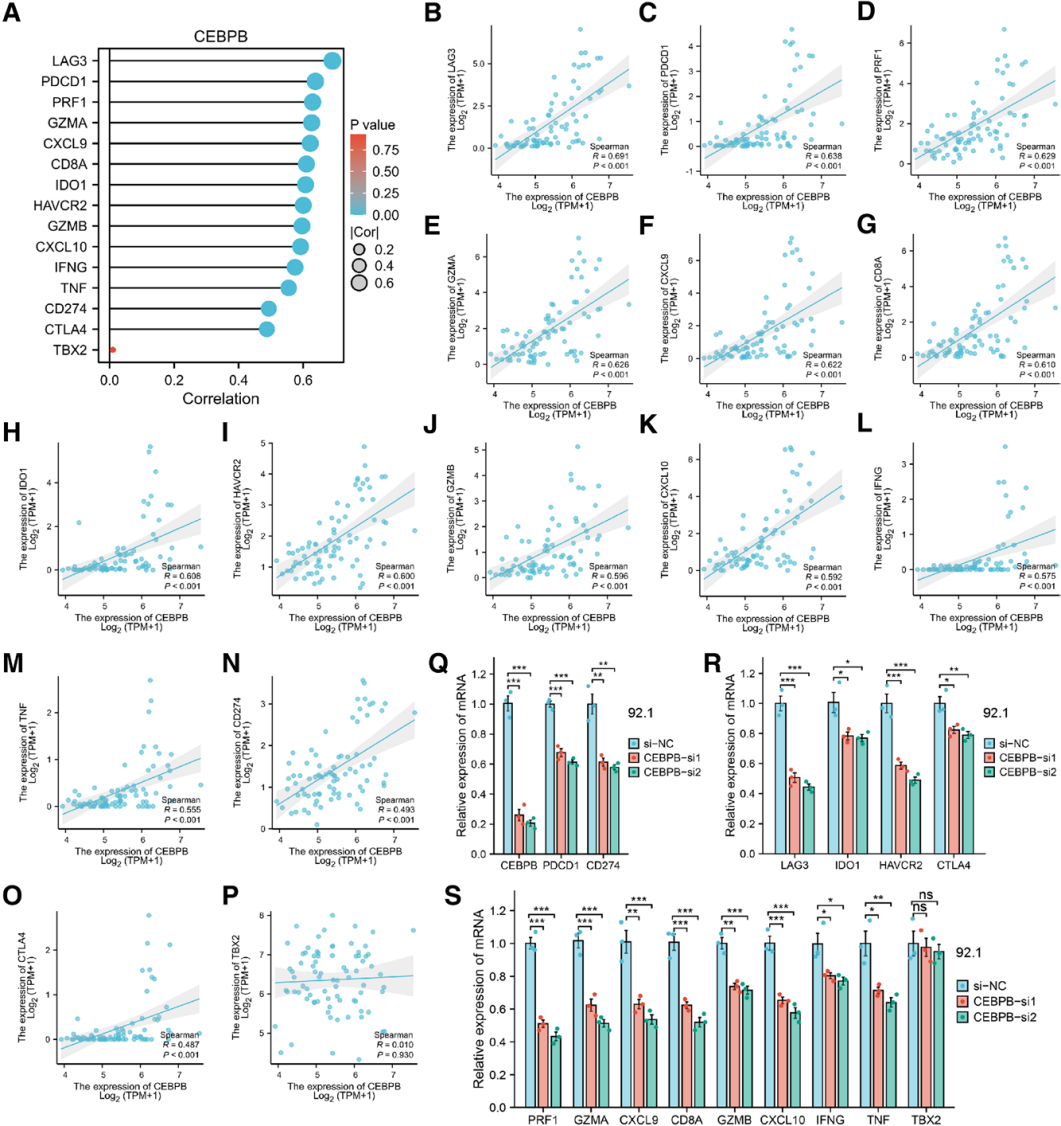
Correlation analysis between CEBPB and common immune checkpoint genes, as well as with immune activation genes. (A) Correlation lollipop chart between CEBPB and immune checkpoint genes, as well as immune activation genes. (B–P) Scatter plot illustrating the correlation between CEBPB and immune checkpoint genes, as well as immune activation genes. Except for a lack of significant correlation with TBX2, CEBPB exhibited a significant positive correlation with all other examined genes. (Q) Expression changes of CEBPB, PD-1 (PDCD1), PDL1 (CD274) following the knockdown of CEBPB in 92.1 cells. (R) Expression changes of LAG3, IDO1, HAVCR2, and CTLA4 following the knockdown of CEBPB in 92.1 cells. (S) Expression changes of CD8A, CXCL10, CXCL9, GZMA, GZMB, IFNG, PRF1, TNF, and TBX2 following the knockdown of CEBPB in 92.1 cells.

We analyzed its correlations with common immune checkpoint genes and immune activation genes. The study found that CEBPB exhibited significant positive correlations with immune checkpoint genes PD-1 (PDCD1), PDL1 (CD274), CTLA4, HAVCR2, IDO1, LAG3, and immune activation genes CD8A, CXCL10, CXCL9, GZMA, GZMB, IFNG, PRF1, and TNF (Fig. 8A–O). However, there was no significant correlation with immune activation gene TBX2 (Fig. 8P). Additionally, we analyzed the overall correlations between 8 immune-related prognostic genes and both immune checkpoint genes and immune activation genes. The results indicated that, with the exception of TBX2, there was generally a positive correlation with the other genes (Fig. S10, Supplemental Digital Content, https://links.lww.com/MD/P27). Subsequently, we further validated the finding in vitro by knocking down CEBPB in the UM cell line 92.1 and assessing the mRNA expression of the related genes. The results demonstrated a significant downregulation in the expression of all the aforementioned genes, except for TBX2 (Fig. 8Q–S). This indicated that CEBPB indeed played a crucial role in the immune activity of UM disease.

### 3.10. Verification of biological function for CEBPB in UM cell lines

**Figure 9.**
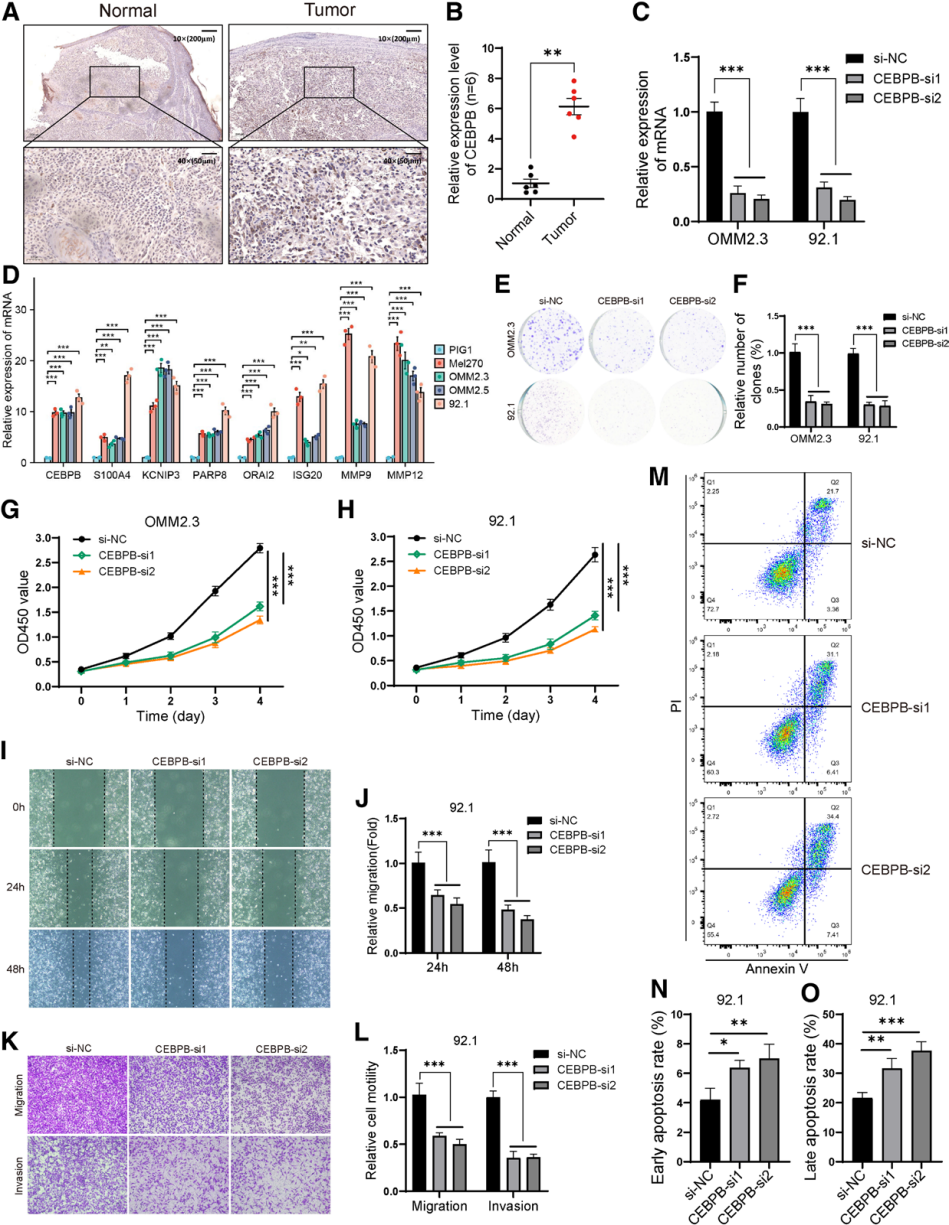
Expression of the prognostic genes and verification of biological function of CEBPB in UM cell lines. (A and B) Immunohistochemical staining and relative expression analysis of CEBPB protein in postoperative tissues of control nevus patients and UM patients. The result indicated a significant increase in the expression of CEBPB in tumor tissues. The images were captured under original magnification × 10 (scale bar, 200 μm) and × 40 (scale bar, 50 μm). (C) Changes in mRNA expression following CEBPB knockdown in OMM2.3 and 92.1 cell lines. (D) Significantly elevated expression of prognostic genes in UM cell lines. (E and F) Colony formation assay indicated that knockdown of CEBPB in the OMM2.3 and 92.1 cells significantly slowed down cell proliferation. (G and H) CCK-8 assay revealed a significant decrease in the activity of OMM2.3 and 92.1 cell lines after the effective knockdown in the expression of CEBPB. (I and J) The scratch wound healing assay indicated that knockdown of CEBPB in the 92.1 cell line significantly reduces cell migration. (K and L) The result of transwell assay indicated that a conspicuously decreased of the migration and invasion ability were observed in 92.1 cells with the knockdown of CEBPB. (M–O) Flow cytometry result indicated that the knockdown of CEBPB increased both early and late apoptosis rates in 92.1 cells. *P < .05, **P < .01, ***P < .001, ****P < .0001. UM = uveal melanoma.

Survival analysis performed on the TCGA-UVM dataset revealed a significant association between increased expression of the CEBPB gene and unfavorable outcomes, including reduced overall survival (OS), disease specific survival, and progression free interval (*P* < .05) (Fig. S4A–C, Supplemental Digital Content, https://links.lww.com/MD/P27). Immunohistochemical analysis of postoperative tissues from UM patients (tumor group) and melanocytic nevus patients (normal group) revealed a significant increase in the expression of CEBPB in the tumor tissues (Fig. 9A and B). In vitro experiments demonstrated elevated levels of CEBPB expression in the UM cell lines (Mel270, OMM2.3, OMM2.5 and 92.1) compared to normal cells PIG1 (Fig. 9D). After knockdown of the CEBPB gene in UM cell lines OMM2.3 and 92.1 following siRNA-mediated targeting, a significant decrease in CEBPB expression was found (Fig. 9C). Functional assays, such as CCK-8 (Fig. 9G and H) and colony formation (Fig. 9E and F) assays, revealed decreased cell viability and proliferative capacity in OMM2.3 and 92.1 cell lines upon CEBPB knockdown. Moreover, scratch wound healing assay (Fig. 9I and J) and transwell assays (Fig. 9K and L) demonstrated a notable reduction in migration and invasion after silencing CEBPB in 92.1 cell line, highlighting its critical role in promoting the survival and aggressive behavior of UM cell lines. Furthermore, flow cytometry result indicated a significant increase in apoptosis, including both early and late apoptosis rates, in 92.1 cells following CEBPB knockdown (Fig. 9M–O). This suggested that knockdown of CEBPB might inhibit cell proliferation and migration by promoting apoptosis in 92.1 cells.

## 4. Discussion

In this study, we had harnessed bioinformatics analysis to delve into the immune landscape of UM by conducting unsupervised clustering of the immune cell infiltration matrix derived from TCGA-UVM dataset. The resultant clusters revealed distinct patterns of immune checkpoint and immune activation related gene expression. Notably, higher expression levels of PD-1, PD-L1, and checkpoint-related genes CTLA4, HAVCR2, IDO1, and LAG3, alongside immune activation related genes CD8A, CXCL10, CXCL9, GZMA, GZMB, IFNG, PRF1, and TNF, were indicative of heightened immune activation in cluster 1. PD-1 and PD-L1 expression was significantly elevated in cluster 1, and immune checkpoint inhibitors targeting them had been applied in the treatment of solid tumors such as cutaneous malignant melanoma, non-small cell lung cancer and colorectal cancer.^[5,14,15]^ Immune checkpoint inhibitors targeting the CTLA4 gene had also shown efficacy in cutaneous malignant melanoma.^[16]^ The study of Durante et al^[17]^ indicated that in UM, tumor-infiltrating immune cells, including CD8 + T cells, predominantly expressed the checkpoint marker LAG3, rather than PD-1 or CTLA4. LAG3 had been identified as a potential candidate for immune checkpoint blockade in high-risk UM patients. In this study, we found significant differences not only in immune infiltration but also in the biological functions exhibited by the DEGs between cluster 1 and cluster 2. Survival analysis combined with patients’ clinical information revealed that patients in cluster 1 had a poorer survival rate.

After conducting module selection on the aforementioned DEGs using WGCNA analysis, we found that the blue module had the highest correlation with cluster 1. GO/KEGG analysis revealed that genes in this module were not only associated with the activation of immune processes and activation of immune cells, but also with cell proliferation and cell growth. The KEGG analysis enriched in the NF-kappa B signaling pathway,^[18]^ JAK-STAT signaling pathway,^[19]^ and TNF signaling pathway,^[20]^ which played crucial roles in the progression of UM disease, as reported by Liu et al, Meng et al, and Damento et al.^[18–20]^

Subsequent univariate Cox regression and LASSO regression analyses were conducted to construct a prognostic prediction model for the genes within the blue module. Utilization of this model based on the genes such as S100A4, KCNIP3, PARP8, ORAI2, MMP12, ISG20, MMP9, and CEBPB to stratify TCGA-UVM samples into high and low-risk groups demonstrated that patients within the high-risk group had poorer prognoses. The model demonstrated an AUC value exceeding 0.8 for predicting the 1-year, 3-year, and 5-year survival rates. Furthermore, the clinical nomogram constructed from the risk score based on the 8 immune-related genes and the clinical characteristics of UM patients underscored its clinical relevance and accuracy in predicting the 1-year, 2-year, and 3-year survival probabilities of patients. Validation with external datasets GSE22138 and GSE84976 corroborated these findings, suggesting the model’s potential to guide clinical decision-making and its broad applicability. Subsequently, we constructed a nomogram incorporating common clinical variables (age, gender, and TNM staging) and the risk score to quantitatively predict the survival rate of patients with UM. Multivariate Cox regression analysis revealed that, after adjusting for these clinical variables, the risk score remained a significant independent prognostic factor. However, due to the lack of detailed treatment information in the TCGA-UVM dataset, we were unable to evaluate the impact of different treatment strategies on the prognostic model. This limitation will be addressed in future studies, in which we will incorporate patients’ treatment history and efficacy data to further optimize the clinical applicability of the model.

The differential gene expression patterns between the high and low-risk groups based on the prognostic model were highly consistent with the gene expression patterns of cluster 1 and cluster 2, indicating the reliability of the model. After conducting GO/KEGG enrichment analysis on the highly expressed genes in the high-risk group, it was found that the majority of biological activities were mainly associated with immune response activation, immune cell activation, cell adhesion, cell proliferation, and cell growth, which was consistent with the enrichment analysis results of the genes in the blue module. The enriched pathways identified through KEGG analysis contained PI3K-Akt signaling pathway, NF-kappa B signaling pathway, JAK-STAT signaling pathway, and TNF signaling pathway. Previous studies had also found that the PI3K-Akt signaling pathway played a significant role in the occurrence and development of UM,^[21,22]^ which was consistent with the results of the above-mentioned study.

There had been numerous reports on the roles played by the 8 prognostic genes (S100A4, KCNIP3, PARP8, ORAI2, MMP12, ISG20, MMP9, CEBPB) in different TMEs of various diseases. Abdelfattah et al identified S100A4 as an immunotherapeutic target for human glioblastoma using single-cell sequencing (scRNA-seq) analysis.^[23]^ Zhou et al identified KCNIP3 as a prognostic biomarker associated with breast cancer recurrence using Logit model analysis.^[24]^ The previous study found that mutations in PARP8 increased the risk of prostate cancer (PCa).^[25]^ Previous research had also shown that ORAI2 promoted the tumorigenicity and metastasis of gastric cancer through the PI3K/Akt signaling pathway.^[26]^ Further research had revealed that MMP12 was involved in the progression of head and neck cancer and induced glycolysis.^[27]^ Huang et al^[28]^ found that as an immune checkpoint, TGF-β1 promoted M2 macrophage polarization and activated the PI3K/mTOR signaling pathway by inhibiting ISG20, rendering ovarian cancer sensitive to cisplatin. The study by Xia et al revealed that a protein complex composed of LCN2, LOXL2, and MMP9 promoted tumor metastasis in esophageal cancer.^[29]^ The study by Li et al^[30]^ found that CEBPB was an immune checkpoint gene in triple-negative breast cancer, and it could control myeloid-derived suppressor cells and tumor immunity through its specific subtypes. In the high-risk group of our study’s test set and validation set, it was observed that the aforementioned 8 prognostic genes showed varying degrees of overexpression, which had an adverse impact on the survival prognosis of UM patients. However, the impact of MMP12 and MMP9 on disease progression could be very limited in UM patients due to their low-expression levels in the tissue. Furthermore, PARP8 and ORAI2 had been experimentally confirmed to be involved in the proliferation, migration, and other biological functions of UM.^[31,32]^ This validated the reliability of the predictive model we constructed in real environments such as UM cells and tissues.

Univariate Cox regression analysis and survival analysis revealed that CEBPB had the highest hazard ratio for influencing the survival rate of UM patients. Moreover, CEBPB exhibited a significant positive correlation with most immune infiltrating cells, as well as with ImmuneScore, ESTIMATEScore, and StromalScore. Therefore, it was considered that CEBPB might promote the progression of UM through the immune pathway. Three different algorithms, CIBERSORT, ssGSEA, and ESTIMATE, yielded similar results. In vitro experiments also confirmed a significant positive correlation between CEBPB and most immune checkpoint genes as well as immune activation genes. These suggested that CEBPB played crucial roles as a potential immune checkpoint gene in the immune microenvironment of UM. As a potential immune checkpoint gene, CEBPB was not only associated with the progression of triple-negative breast cancer^[30]^ but also promoted the occurrence and drug resistance of gastric cancer^[33]^ and ovarian cancer,^[34]^ and played a role in the progression of PCa.^[35]^ Similarly to the aforementioned results, our current study had also revealed that CEBPB could participate in the occurrence and development of UM through the immune pathway, leading to lower survival rates in patients with UM. In particular, the in vitro validation of CEBPB biological function in the UM cell lines OMM2.3 and 92.1 positioned CEBPB as a promising molecular target for UM therapy. The exploration of CEBPB molecular mechanisms would be essential to fully leverage its therapeutic potential, which was an aspect that our future research endeavors will aim to explore. Additionally, given its strong correlation with immune checkpoint genes and immune cell infiltration, CEEBPB is likely to play a role in immune escape. Future studies are required to further explore its specific mechanism in immune escape of UM.

Although our study demonstrated excellent predictive performance and the results showed high reliability and stability, certain limitations still exist. Firstly, our research primarily relied on publicly available datasets from the TCGA and GEO databases. Although external validation was conducted in 2 independent datasets, the generalization ability of the model still needs to be further verified in larger and more diverse cohorts. In the future, we plan to collect samples from UM patients across multiple centers and, in combination with prospective studies, further validate the clinical applicability of this model. Additionally, we will also explore the predictive value of this model under different immunotherapy regimens to enhance its potential for application in clinical practice. Moreover, although we confirmed the impact of the CEBPB gene on the biological functions in UM cell lines, the specific molecular mechanisms remain unclear and require further experimental research.

## 5. Conclusion

Our study presented an immune-related prognostic prediction model for UM. Furthermore, this model demonstrated a strong predictive accuracy for the prognosis of UM patients. Eight genes in the prognostic model showed increased expression in UM cells, with CEBPB potentially promoting UM cell proliferation and inhibiting apoptosis, which could play an important role in the progression of UM disease.

## Acknowledgments

We extend our appreciation to the repositories of the GEO and TCGA for making UM sample datasets and clinical information accessible at no cost. Our thanks also go to all contributors whose efforts made the publication of this paper possible.

## Author contributions

**Conceptualization:** Yulin Tao, Qiong Zhou.

**Data curation:** Yulin Tao, Haibo Zhu.

**Formal analysis:** Yulin Tao, Yirui Peng.

**Funding acquisition:** Qiong Zhou, Jun Ouyang.

**Investigation:** Yirui Peng, Haibo Zhu, Minqi Xiong.

**Methodology:** Yirui Peng, Minqi Xiong, Jun Ouyang.

**Project administration:** Qiong Zhou.

**Resources:** Yirui Peng, Haibo Zhu.

**Software:** Yirui Peng, Minqi Xiong.

**Supervision:** Yulin Tao, Qiong Zhou, Jun Ouyang.

**Validation:** Yulin Tao.

**Visualization:** Yirui Peng, Haibo Zhu, Minqi Xiong.

**Writing – original draft:** Yulin Tao.

**Writing – review & editing:** Qiong Zhou, Jun Ouyang.

## Supplementary Material



## References

[1] Jager MJ Shields CL Cebulla CM. Uveal melanoma. Nat Rev Dis Primers. 2020;6:24. 32273508 10.1038/s41572-020-0158-0

[2] Smit KN Jager MJ de Klein A Kiliҫ E. Uveal melanoma: towards a molecular understanding. Prog Retin Eye Res. 2020;75:100800. 31563544 10.1016/j.preteyeres.2019.100800

[3] Fu Y Xiao W Mao Y. Recent advances and challenges in uveal melanoma immunotherapy. Cancers (Basel). 2022;14:3094. 35804863 10.3390/cancers14133094 PMC9264803

[4] Lamas NJ Martel A Nahon-Esteve S. Prognostic biomarkers in uveal melanoma: the status quo, recent advances and future directions. Cancers (Basel). 2021;14:96. 35008260 10.3390/cancers14010096 PMC8749988

[5] Zhang H Liu L Liu J. Roles of tumor-associated macrophages in anti-PD-1/PD-L1 immunotherapy for solid cancers. Mol Cancer. 2023;22:58. 36941614 10.1186/s12943-023-01725-x PMC10029244

[6] Berman DM Bell JI. Redirecting polyclonal T cells against cancer with soluble T-cell receptors. Clin Cancer Res. 2023;29:697–704. 36255733 10.1158/1078-0432.CCR-22-0028 PMC9932579

[7] Zeng D Ye Z Shen R. IOBR: Multi-omics immuno-oncology biological research to decode tumor microenvironment and signatures. Front Immunol. 2021;12:687975. 34276676 10.3389/fimmu.2021.687975 PMC8283787

[8] Wilkerson MD Hayes DN. ConsensusClusterPlus: a class discovery tool with confidence assessments and item tracking. Bioinformatics (Oxford, England). 2010;26:1572–3. 20427518 10.1093/bioinformatics/btq170 PMC2881355

[9] Zhang X Shi M Chen T Zhang B. Characterization of the immune cell infiltration landscape in head and neck squamous cell carcinoma to aid immunotherapy. Mol Ther Nucleic Acids. 2020;22:298–309. 33230435 10.1016/j.omtn.2020.08.030 PMC7522342

[10] Wu T Hu E Xu S. clusterProfiler 4.0: A universal enrichment tool for interpreting omics data. Innovation (Camb). 2021;2:100141. 34557778 10.1016/j.xinn.2021.100141 PMC8454663

[11] Beyene KM El Ghouch A. Time-dependent ROC curve estimation for interval-censored data. Biom J. 2022;64:1056–74. 35523738 10.1002/bimj.202000382

[12] Gustavsson EK Zhang D Reynolds RH Garcia-Ruiz S Ryten M. ggtranscript: an R package for the visualization and interpretation of transcript isoforms using ggplot2. Bioinformatics. 2022;38:3844–6. 35751589 10.1093/bioinformatics/btac409 PMC9344834

[13] Ning W Wei Y Gao L. HemI 2.0: an online service for heatmap illustration. Nucleic Acids Res. 2022;50:W405–11. 35670661 10.1093/nar/gkac480 PMC9252725

[14] Bao Y Zhai J Chen H. Targeting m6A reader YTHDF1 augments antitumour immunity and boosts anti-PD-1 efficacy in colorectal cancer. Gut. 2023;72:1497–509. 36717220 10.1136/gutjnl-2022-328845 PMC10359538

[15] Du F Yang LH Liu J. The role of mitochondria in the resistance of melanoma to PD-1 inhibitors. J Transl Med. 2023;21:345. 37221594 10.1186/s12967-023-04200-9 PMC10207731

[16] Buchbinder EI Desai A. CTLA-4 and PD-1 pathways: similarities, differences, and implications of their inhibition. Am J Clin Oncol. 2016;39:98–106. 26558876 10.1097/COC.0000000000000239 PMC4892769

[17] Durante MA Rodriguez DA Kurtenbach S. Single-cell analysis reveals new evolutionary complexity in uveal melanoma. Nat Commun. 2020;11:496. 31980621 10.1038/s41467-019-14256-1 PMC6981133

[18] Liu N Sun Q Chen J. MicroRNA-9 suppresses uveal melanoma cell migration and invasion through the NF-κB1 pathway. Oncol Rep. 2012;28:961–8. 22825752 10.3892/or.2012.1905

[19] Meng S Zhu T Fan Z. Integrated single-cell and transcriptome sequencing analyses develops a metastasis-based risk score system for prognosis and immunotherapy response in uveal melanoma. Front Pharmacol. 2023;14:1138452. 36843929 10.3389/fphar.2023.1138452 PMC9947539

[20] Damento GM Pulido JS Abbott BA Hodge DO Dalvin LA. TNF-Alpha inhibition and other immunosuppressants in the development of uveal and cutaneous melanoma. Mayo Clin Proc. 2019;94:1287–95. 31272570 10.1016/j.mayocp.2018.11.033

[21] Pérez-Pérez M Agostino A de Sola-Llamas CG. Next-generation sequencing of uveal melanoma with clinical and histological correlations: prognostic value of new mutations in the PI3K/AKT/mTOR pathway. Clin Exp Ophthalmol. 2023;51:822–34. 37803816 10.1111/ceo.14302

[22] Geng Y Geng Y Liu X. PI3K/AKT/mTOR pathway-derived risk score exhibits correlation with immune infiltration in uveal melanoma patients. Front Oncol. 2023;13:1167930. 37152048 10.3389/fonc.2023.1167930 PMC10157141

[23] Abdelfattah N Kumar P Wang C. Single-cell analysis of human glioma and immune cells identifies S100A4 as an immunotherapy target. Nat Commun. 2022;13:767. 35140215 10.1038/s41467-022-28372-y PMC8828877

[24] Zhou X Xiao C Han T. Prognostic biomarkers related to breast cancer recurrence identified based on Logit model analysis. World J Surg Oncol. 2020;18:254. 32977823 10.1186/s12957-020-02026-z PMC7519567

[25] Alfahed A Ebili HO Almoammar NE. Prognostic values of gene copy number alterations in prostate cancer. Genes (Basel). 2023;14:956. 37239316 10.3390/genes14050956 PMC10217731

[26] Wu S Chen M Huang J. ORAI2 promotes gastric cancer tumorigenicity and metastasis through PI3K/Akt signaling and MAPK-dependent focal adhesion disassembly. Cancer Res. 2021;81:986–1000. 33310726 10.1158/0008-5472.CAN-20-0049

[27] Huang TL Chang CR Chien CY. DRP1 contributes to head and neck cancer progression and induces glycolysis through modulated FOXM1/MMP12 axis. Mol Oncol. 2022;16:2585–606. 35313071 10.1002/1878-0261.13212 PMC9251862

[28] Wu J Jiang L Wang S Peng L Zhang R Liu Z. TGF β1 promotes the polarization of M2-type macrophages and activates PI3K/mTOR signaling pathway by inhibiting ISG20 to sensitize ovarian cancer to cisplatin. Int Immunopharmacol. 2024;134:112235. 38761779 10.1016/j.intimp.2024.112235

[29] Xia Q Du Z Chen M. A protein complex of LCN2, LOXL2 and MMP9 facilitates tumour metastasis in oesophageal cancer. Mol Oncol. 2023;17:2451–71. 37753805 10.1002/1878-0261.13529 PMC10620126

[30] Li W Tanikawa T Kryczek I. Aerobic glycolysis controls myeloid-derived suppressor cells and tumor immunity via a specific CEBPB isoform in triple-negative breast cancer. Cell Metab. 2018;28:87–103.e6. 29805099 10.1016/j.cmet.2018.04.022 PMC6238219

[31] Li X Kang J Yue J. Identification and validation of immunogenic cell death-related score in uveal melanoma to improve prediction of prognosis and response to immunotherapy. Aging (Milano). 2023;15:3442–64. 10.18632/aging.204680 PMC10449274 37142279

[32] Huang W Yang F Zhang Y Fang Q Lai Y Lan Y. A newly established cuproptosis-related gene signature for predicting prognosis and immune infiltration in uveal melanoma. Int J Mol Sci . 2023;24:11358. 37511120 10.3390/ijms241411358 PMC10379443

[33] Wu H Liu B Chen Z Li G Zhang Z. MSC-induced lncRNA HCP5 drove fatty acid oxidation through miR-3619-5p/AMPK/PGC1α/CEBPB axis to promote stemness and chemo-resistance of gastric cancer. Cell Death Dis. 2020;11:233. 32300102 10.1038/s41419-020-2426-z PMC7162922

[34] Wang H Zhou Y Zhang S Qi YA Wang M. PRPF6 promotes metastasis and paclitaxel resistance of ovarian cancer via SNHG16/CEBPB/GATA3 axis. Oncol Res. 2021;29:275–89. 37303939 10.32604/or.2022.03561 PMC10208018

[35] Chen S Lu K Hou Y. YY1 complex in M2 macrophage promotes prostate cancer progression by upregulating IL-6. J ImmunoTher Cancer. 2023;11:e006020. 37094986 10.1136/jitc-2022-006020 PMC10152059

