# Supplementary material for: Construction of an immune-related prognostic model and functional analysis of CEBPB in uveal melanoma: A STROBE-compliant observational study
Source: Medicine (Baltimore). 2025 Jun 20;104(25):e42574. doi: 10.1097/MD.0000000000042574 (PMC12187318; doi:10.1097/MD.0000000000042574)
Supplement: Supplementary file 1 [file medi-104-e42574-s001.pdf]

Supplementary materials and methods

1. TIDE analysis

Utilizing the TCGA-UVM dataset, we calculated TIDE scores for high-risk and low-risk groups to predict the interaction patterns between tumor cells and immune cells.

2. Cell culture and transfection

The normal PIG1 cell line was obtained from Otwo Biotech Inc. (ShenZhen, China). The human UM cell line 92.1 was sourced from Procell (Wuhan, China). The OMM2.3, OMM2.5, and Mel270 cell lines were obtained from Cellcook Biotech Co., Ltd. (Guangzhou, China). PIG1 cells were cultivated in DMEM medium (Gibco, USA), while the cell lines about Mel270, OMM2.3, OMM2.5, and 92.1 were grown in RPMI 1640 medium (Gibco, USA). Both medium were supplemented with 10% sterilized fetal bovine serum (FBS, Gibco, USA) and 1% antibiotic-antimycotic solution (Procell, Wuhan, China). All cell lines were maintained under optimal culture conditions at 37℃ with 5% CO_2. For gene silencing experiments, synthetic small interfering RNAs (siRNAs) targeting the CEBPB gene were purchased from Hanbio Tech (Shanghai, China). The transfection of siRNAs into the cell lines was performed using the Lipofectamine2000 reagent (Thermo Fisher Scientific, USA), following the guidelines provided by Thermo Scientific. The detailed sequences of the siRNAs used can be found in Table S1.

3. RNA isolation and quantitative real-time PCR (qRT-PCR)

RNA was isolated utilizing Trizol Reagent (Tiangen Biotech Co., Ltd., Beijing, China) and subsequently purified employing chloroform, isopropanol, and ethanol. Subsequent cDNA synthesis was executed utilizing the FastKing RT Kit (Tiangen Biotech), and the quantification of gene expression was carried out through SYBR Green-based qPCR (Tiangen Biotech) on an ABI Step One system (USA), with normalization to GAPDH utilizing the -2△△Ct method. Primer sequences could be found in Supplementary Table S2.

4. Cell viability and proliferation assay

The viability assessment of OMM2.3 and 92.1 cell lines was performed using the CCK-8 assay (Yeasen, Shanghai, China). Approximate 7,000 OMM2.3 or 92.1 cells were seeded in a well of 96-well plate, followed by the addition of RPMI 1640 medium (100 μl). Subsequently, the CCK-8 solution was added to the wells, and the plate was incubated at 37°C for 2 hours. Absorbance readings at 450 nm were acquired using an enzyme-linked detection instrument (TECAN, Männedorf, Switzerland) at 24-hour intervals for a total of four days. Clonogenic assay was employed to assess cell proliferation. OMM2.3 and 92.1 cells were seeded at a density of 1,000 cells per well in 6-well plate containing RPMI 1640 (2 ml). The plates were then incubated at 37 °C for a period of 10 days. Then, the cells were fixed using 4% paraformaldehyde and stained with crystal violet. The resulting colonies were captured using a digital scanner (Hewlett-Packard, USA) and quantitatively analyzed using Image J software.

5. Cell migration and invasion assay

To investigate the migratory potential of 92.1 cells in relation to the expression of CEBPB, we conducted scratch assay. Once the cells reached 90-100% confluence in serum-free medium, a

scratch was created. The cells were washed and imaged at 0, 24, and 48 hours after scratch. The migration process was quantitatively analyzed using Image J software.

We also assessed the migratory and invasive capabilities of 92.1 cells using a transwell system (Corning, USA). In migration assays, cells (1×10^5) suspended in RPMI 1640 medium containing 10% FBS were placed in the upper chamber, while the lower chamber contained medium supplemented with 20% FBS. For invasion assays, the filters were coated with Matrigel (Corning, USA). Following a 24-hour incubation at 37 °C, the cells on the filters were fixed, stained with crystal violet, and non-migratory cells were removed. The migrated or invaded cells on the bottom surface were then counted using light microscopy (Olympus, Japan) and Image J software.

6. Immunohistochemistry

Under the conditions in accordance with the Helsinki Declaration and approved by the ethics committee of the first affiliated hospital of nanchang university, clinical tissue samples were obtained from 6 postoperative UM patients and 6 postoperative melanocytic nevus patients. Approval number: 2023-CDYFYYLK-11-019. Clinical and pathological information for patients with UM was presented in Table S3. The tissues were fixed with paraformaldehyde, embedded in paraffin, and then subjected to deparaffinization. Antigen retrieval was performed using a sodium citrate solution (Servicebio, Wuhan, China) at 100°C, followed by endogenous peroxidase blocking with 3% hydrogen peroxide (H2O2, Beyotime, Shanghai, China). The sections were then incubated with the primary antibody (CEBPB, Abcam, 1:500) at 4°C for 14 hours, followed by the secondary antibody conjugated with horseradish peroxidase (Beyotime, Shanghai, China) at room temperature for 1 hour. Immunostaining was performed using DAB chromogenic solution (Solarbio, Beijing, China) under dark conditions, followed by counterstaining with hematoxylin to visualize the cell nucleus. Photographs of three fields of view were captured for each section under a microscope (Olympus, Japan).

7. Flow cytometry

Following siRNA transfection, cells were digested with trypsin and collected, then washed twice with precooled phosphate-buffered saline (PBS). The cells were counted and diluted to a concentration of 1×10^5 cells/ml. Cells were resuspended in 100 μl of binding buffer and stained with 5 μl of Annexin V-FITC and 10 μl of propidium iodide at room temperature for 15 minutes, followed by 400 μl of binding buffer added to resuspend the cells. The rate of apoptotic cells was assessed with flow cytometry (Agilent NovoCyte flow cytometer, USA).
