# Supplementary material for: Construction of an immune-related prognostic model and functional analysis of CEBPB in uveal melanoma: A STROBE-compliant observational study
Source: Medicine (Baltimore). 2025 Jun 20;104(25):e42574. doi: 10.1097/MD.0000000000042574 (PMC12187318; doi:10.1097/MD.0000000000042574)
Supplement: Supplementary file 2 [file medi-104-e42574-s002.pdf]

Table S1. The detailed sequences of siRNAs in this study.

siRNAs		Sequences
si-NC	Sense	5' UUCUCCGAACGUGUCACGUTT 3'
	Antisense	5' ACGUGACACGUUCGGAGAATT 3'
CEBPB-si1	Sense	5' GGAACUUGUUCAAGCAGCUTT 3'
	Antisense	5' AGCUGCUUGAACAAGUUCCTT 3'
CEBPB-si2	Sense	5' AGCACAGCGACGAGUACAATT 3'
	Antisense	5' UUGUACUCGUCGCUGUGCUTT 3'

Table S2. Primer sequences used in this study.

Primers		Sequences
GAPDH	Sense	5' GGAAGCTTGTCATCAATGGAAATC 3'
	Antisense	5' TGATGACCCTTTTGGCTCCC 3'
CEBPB	Sense	5' GGACAAGCACAGCGACGAGTA 3'
	Antisense	5' TTGAACAAGTTCCGCAGGGT 3'
PDCD1	Sense	5' GAGGGAATGCGTATTTTGGGT 3'
	Antisense	5' AGGTTGTTCTTGTGTCACCTG 3'
CD274	Sense	5' TGGCATTTGCTGAACGCATTT 3'
	Antisense	5' TGCAGCCAGGTCTAATTGTTTT 3'
CTLA4	Sense	5' GCCCTGCACTCTCCTGTTTTT 3'
	Antisense	5' GGTTGCCGCACAGACTTCA 3'
HAVCR2	Sense	5' AGACAGTGGGATCTACTGCTG 3'
	Antisense	5' CCTGGTGGTAAGCATCCTTGG 3'
IDO1	Sense	5' TCTCATTTCGTGATGGAGACTGC 3'
	Antisense	5' GTGTCCCGTTCTTGCATTTGC 3'
LAG3	Sense	5' GCGGGGACTTCTCGCTATG 3'
	Antisense	5' GGCTCTGAGAGATCCTGGGG 3'
CD8A	Sense	5' ATGGCCTTACCAGTGACCG 3'
	Antisense	5' AGGTTCCAGGTCCGATCCAG 3'
CXCL10	Sense	5' GTGGCATTCAAGGAGTACCTC 3'
	Antisense	5' TGATGGCCTTCGATTCTGGATT 3'
CXCL9	Sense	5' CCAGTAGTGAGAAAGGGTCGC 3'
	Antisense	5' AGGGCTTGGGGCAAATTGTT 3'
GZMA	Sense	5' TCTCTCTCAGTTGTCGTTTCTCT 3'
	Antisense	5' GCAGTCAACACCCAGTCTTTTG 3'
GZMB	Sense	5' CCCTGGGAAAACACTCACACA 3'
	Antisense	5' GCACAACTCAATGGTACTGTCG 3'
IFNG	Sense	5' AGCAGGAAGTCGATTATGATCCC 3'
	Antisense	5' CTGGCACTGAATCTCGTCACA 3'

PRF1	Sense	5' GACTGCCTGACTGTCGAGG 3'
	Antisense	5' TCCCGGTAGGTTTGGTGGAA 3'
TNF	Sense	5' CGGCTACCTAGTCTACGCC 3'
	Antisense	5' AAGTCGCCGCCAATGTTGA 3'
TBX2	Sense	5' CCCCTTCAAGGTGCGAGTC 3'
	Antisense	5' TCAGCGGCTACAATGTCCATC 3'

Table S3. Clinical and pathological information for patients with UM.

Clinical sample characteristics	
age (years)	
Range	33 ~ 73
Mean±SD	54.17±14.03
gender	
Male	4
Female	2
T stage	
T1	1
T2	2
T3	2
T4	1
N stage	
N0	6
M stage	
M0	6
Clinical stage	
IIA	5
IIB	1
Metastasis	
yes	2
no	4
