# Supplementary material for: Construction of an immune-related prognostic model and functional analysis of CEBPB in uveal melanoma: A STROBE-compliant observational study
Source: Medicine (Baltimore). 2025 Jun 20;104(25):e42574. doi: 10.1097/MD.0000000000042574 (PMC12187318; doi:10.1097/MD.0000000000042574)
Supplement: Supplementary file 3 [file medi-104-e42574-s003.pdf]

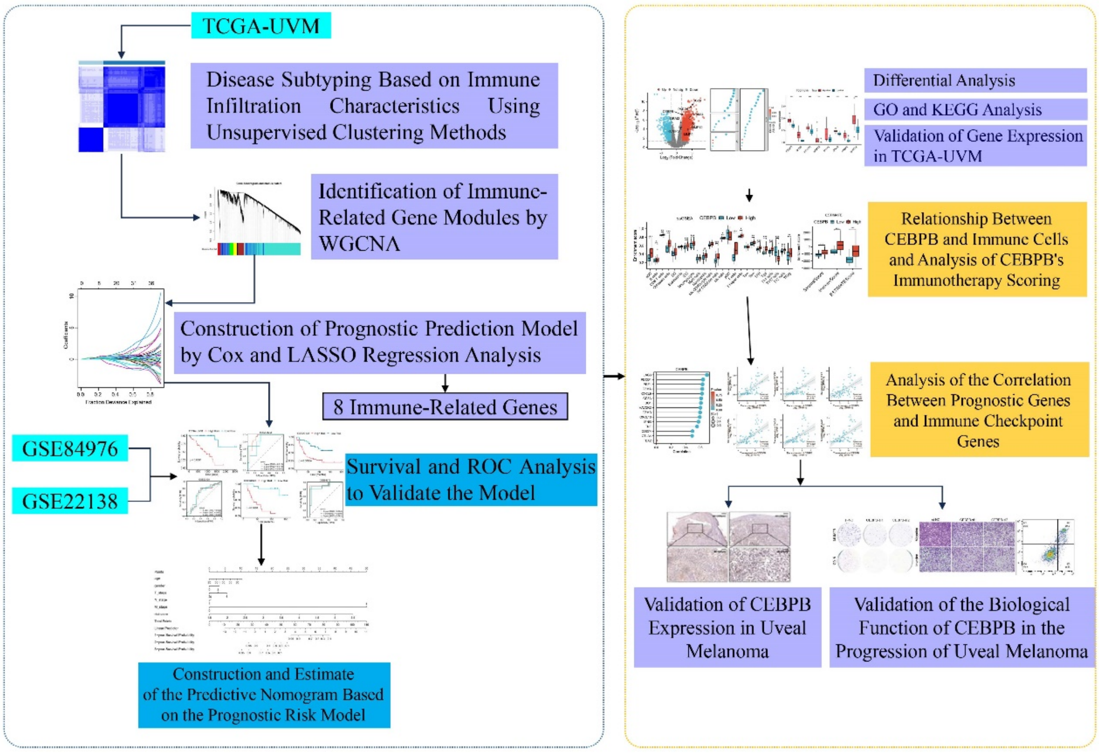

Fig. S1. Overall analysis flow chart.

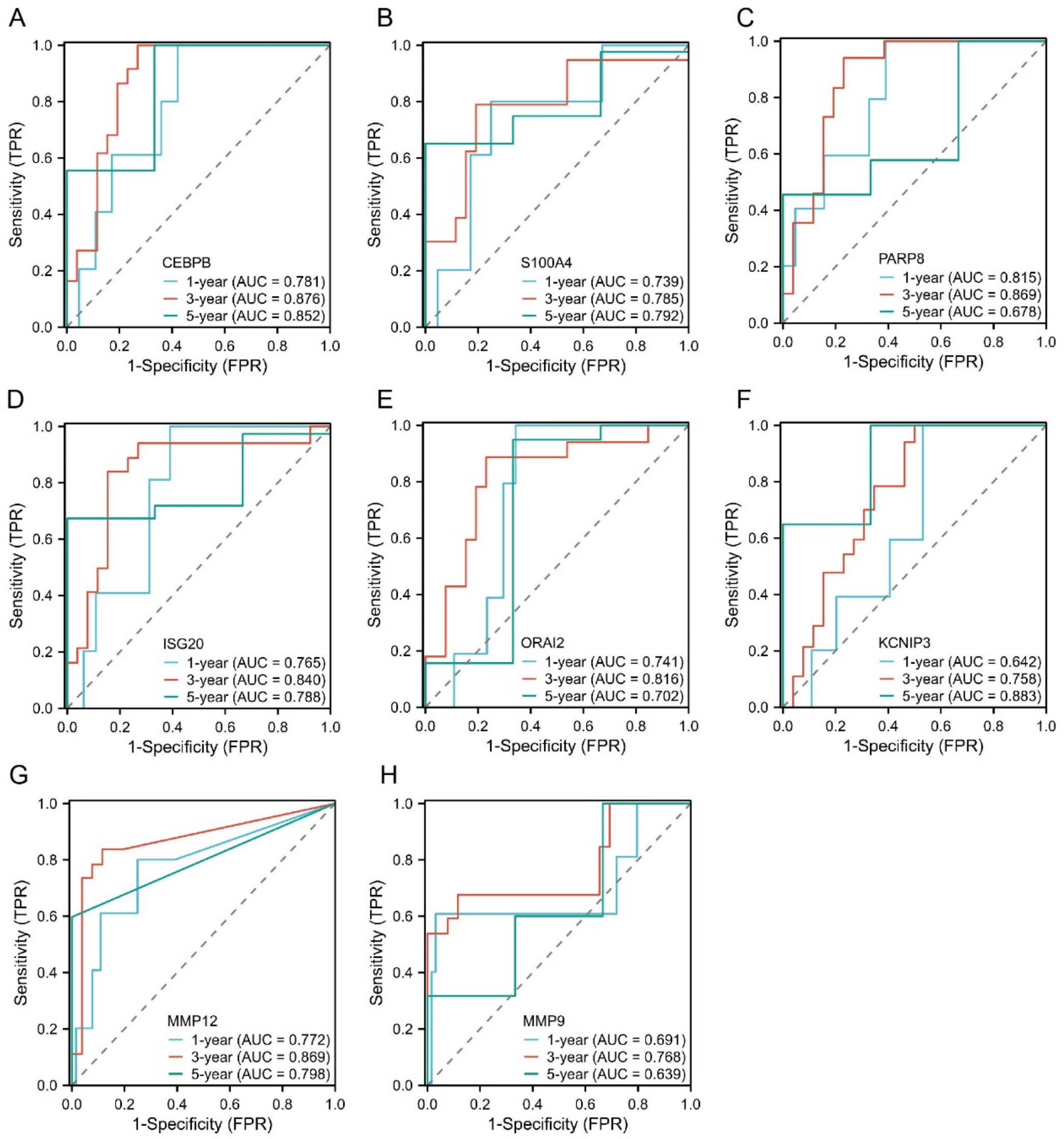

Fig. S2. ROC curves of 8 prognostic genes (S100A4, KCNIP3, PARP8, ORAI2, MMP12, ISG20, MMP9, CEBPB) predicting patient survival accuracy were presented. The results indicated that the predictive performance of each gene individually was inferior to the prognostic model constructed by the 8 prognostic genes. (ROC: receiver operating characteristic curves).

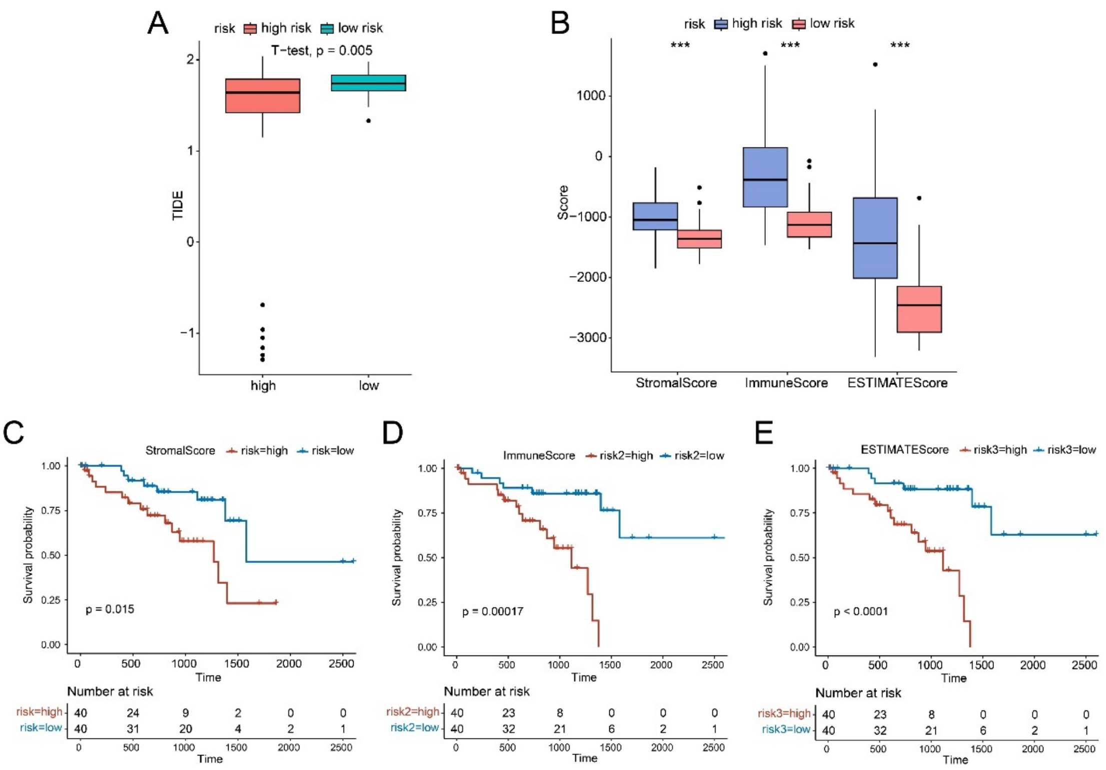

Fig. S3. Immunotherapy and immune scoring for the prognostic model. (A) Comparison of TIDE scores between high and low-risk groups in UM patients. (B) Differences in StromalScore, ImmuneScore, and ESTIMATEScore between high and low-risk groups in UM patients. (C) In the TCGA-UVM dataset, the KM survival curves for StromalScore. (D) The KM survival curves for ImmuneScore. (E) The KM survival curves for ESTIMATEScore. (red represents the high score group, blue represents the low score group). (TCGA-UVM: the cancer genome atlas-uveal melanoma; UM: uveal melanoma; KM: Kaplan-Meier).

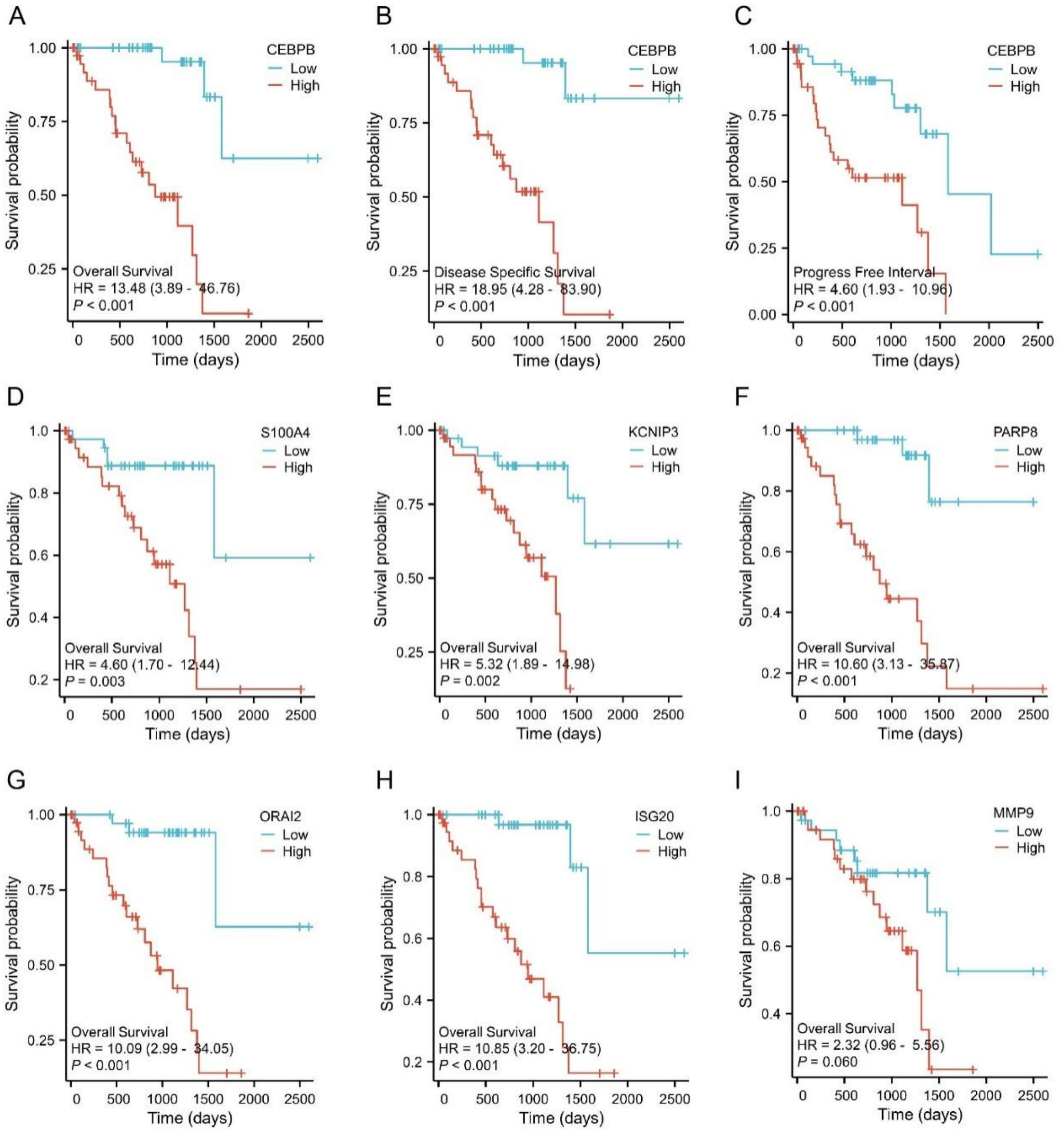

Fig. S4. Survival curves of prognostic genes in high and low expression groups of TCGA-UVM patients. Based on the median expression value of the genes, TCGA-UVM patients were divided into high and low expression groups. (A-C) The impact of high CEBPB expression on the survival of TCGA-UVM patients. Panels A-C respectively illustrated the impact of high CEBPB expression on the OS, DSS and PFI of TCGA-UVM patients. The result suggested that high CEBPB expression significantly reduced the survival period of UM patients. (D-H) Panels D-H respectively showed the impact of high expression of S100A4, KCNIP3, PARP8, ORAI2, and ISG20 genes on the OS of UM patients. (I) Except for MMP9, high expression of the other five prognostic genes significantly reduced the survival rate of UM patients. The low expression levels of MMP12 in TCGA-UVM patients prevented the construction of a survival curve. (TCGA-UVM: the cancer genome atlas-uveal melanoma; OS: overall survival; DSS: disease specific survival; PFI: progress free interval).

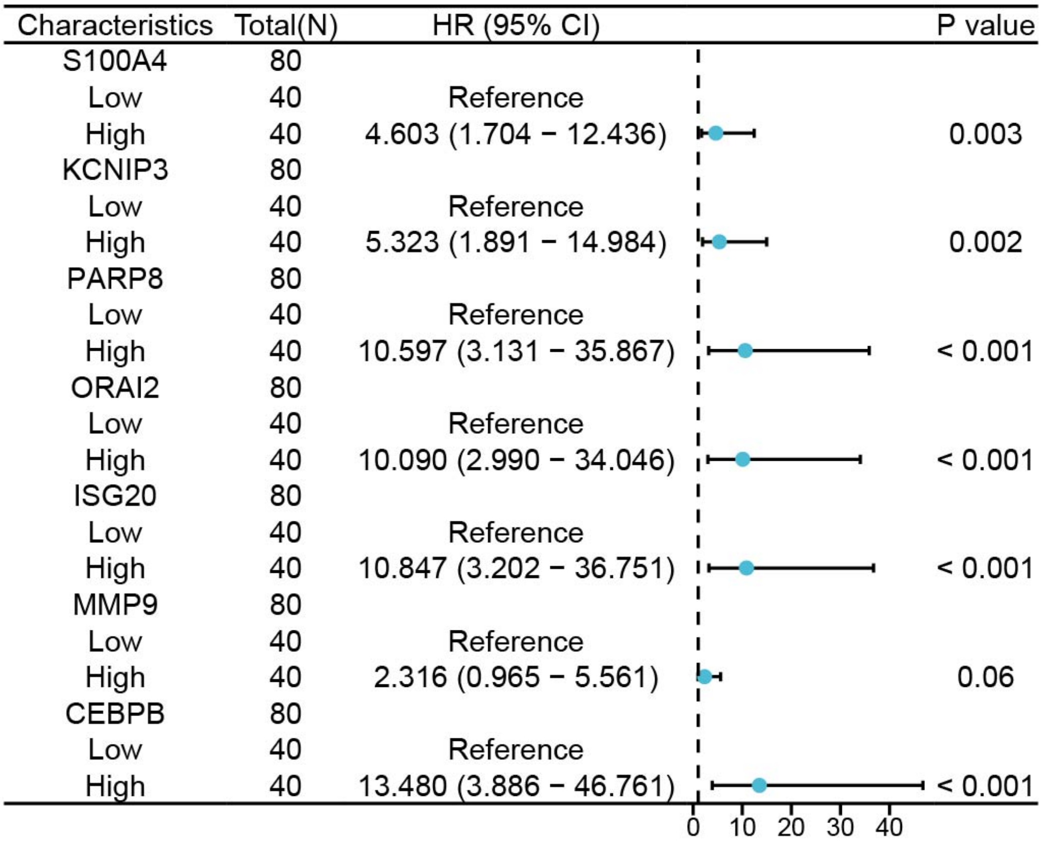

Fig. S5. Univariate Cox regression analysis of 7 prognostic genes (S100A4, KCNIP3, PARP8, ORAI2, ISG20, MMP9, CEBPB) based on the TCGA-UVM dataset. The results indicated that S100A4, KCNIP3, PARP8, ORAI2, ISG20, and CEBPB were poor prognostic risk factors for UM patients. Specifically, CEBPB posted the highest risk of reducing the survival rate in UM patients. (TCGA-UVM: the cancer genome atlas-uveal melanoma; UM: uveal melanoma).

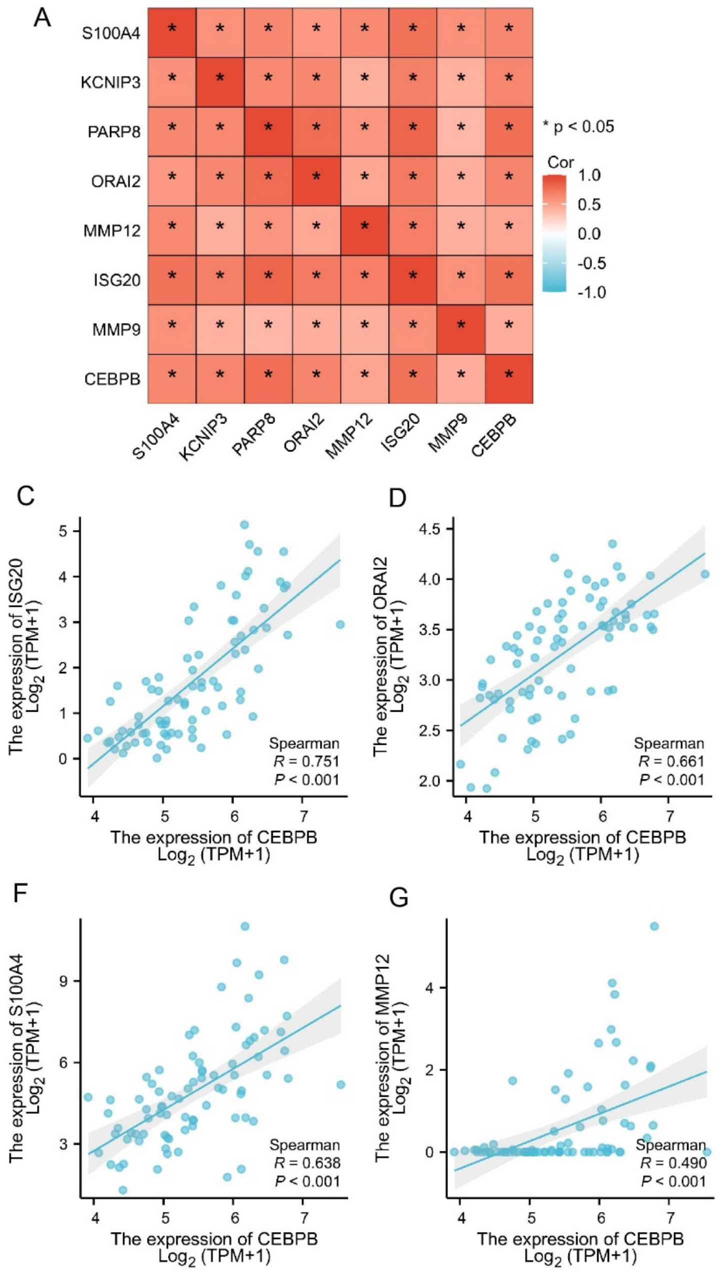

Fig. S6. Analysis of correlations among prognostic genes. (A) Correlation heatmap of the eight genes in the prognostic model. There was a significant positive correlation among the eight genes. (B-H) Scatter plot showed the correlation of CEBPB with the other 7 genes (S100A4, KCNIP3, PARP8, ORAI2, MMP12, ISG20, MMP9). The results demonstrated a positive correlation with all genes, with the highest correlation observed with PARP8.

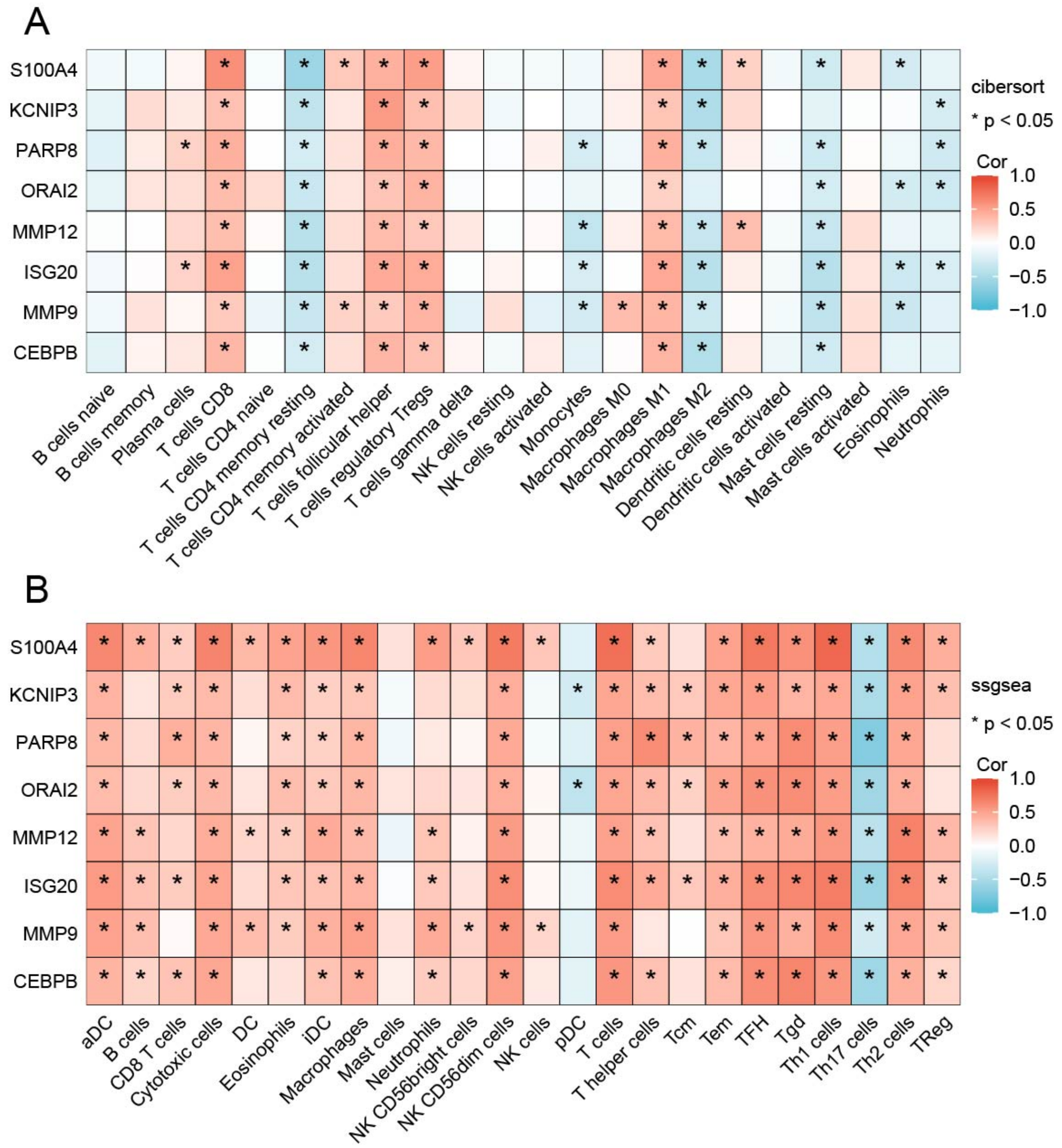

Fig. S7. Correlation heatmap of prognostic genes with immune cells. (A) The correlation heatmap displayed significant correlations of the 8 prognostic genes with 7 immune cells to varying degrees. (B) The correlation heatmap showed significant correlations of the 8 prognostic genes with 18 immune cells to varying degrees.

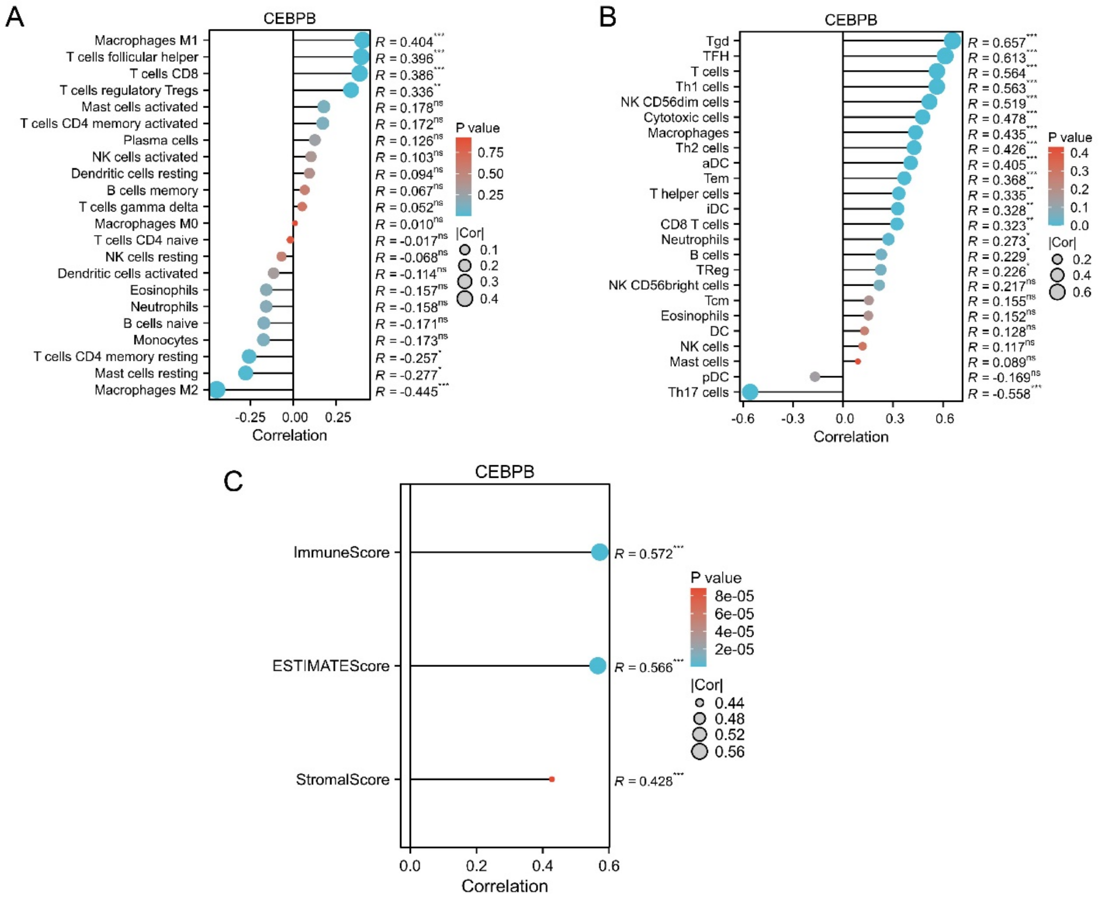

Fig. S8. Lollipop plot illustrating the correlations between CEBPB and immune infiltrating cells, as well as immune-related scores, as calculated by the CIBERSORT, ssGSEA, and ESTIMATE algorithms. (A) Results from the CIBERSORT algorithm indicated that CEBPB was significantly positively correlated with Macrophages M1, T cells follicular helper, T cells CD8, and T cells regulatory Tregs, while showing significant negative correlations with Mast cells resting, T cells CD4 memory resting, and Macrophages M2. (B) Results from the ssGSEA algorithm indicated that CEBPB was significantly positively correlated with most immune cells, with the notable exception of a significant negative correlation with Th17 cells. (C) Results from the ESTIMATE algorithm indicated that CEBPB exhibited significant positive correlations with ImmuneScore, ESTIMATEScore, and StromalScore.

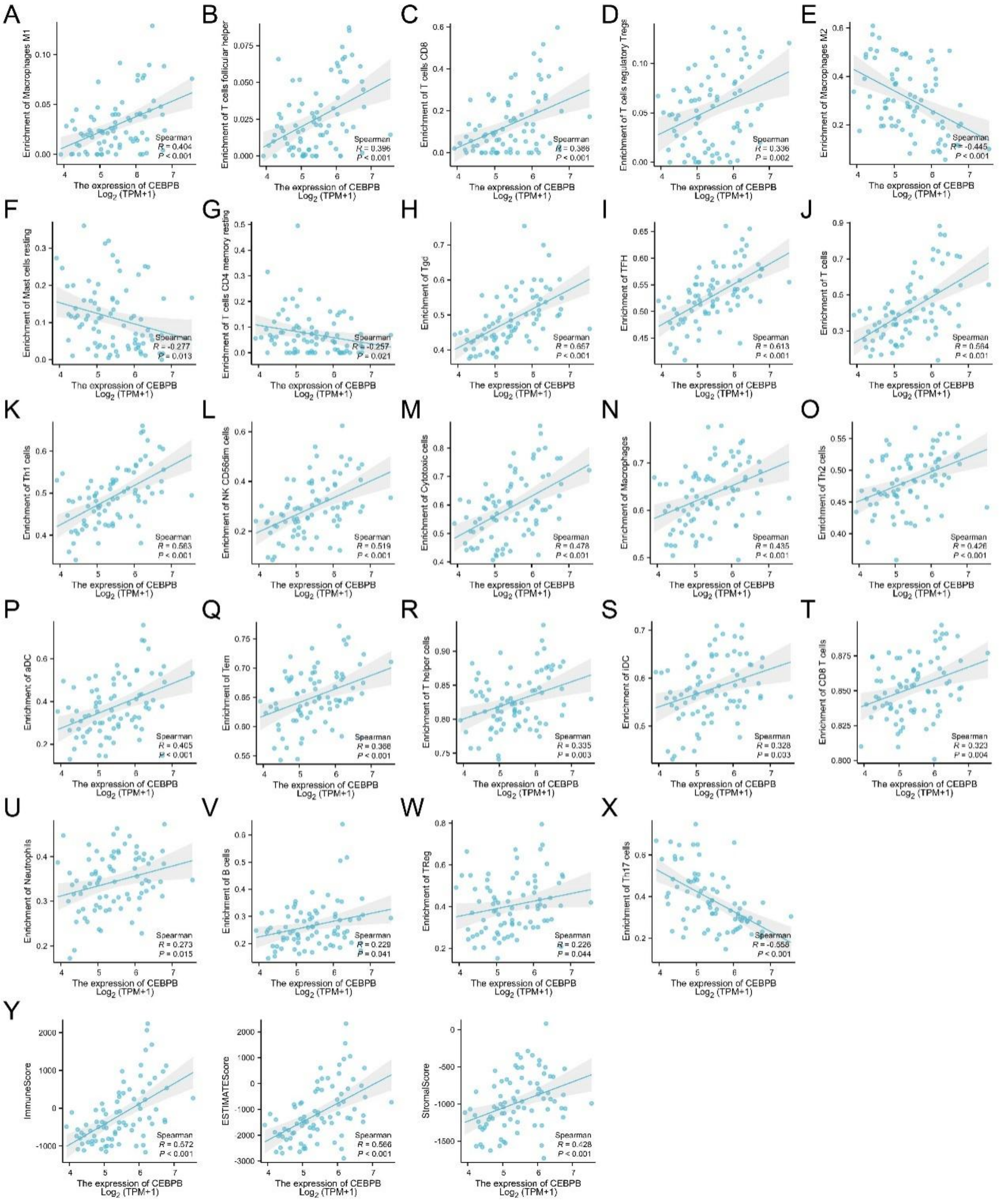

Fig. S9. Correlation of CEBPB with immune cells and immune scoring. (A-G) The results derived from the CIBERSORT algorithm suggested that CEBPB exhibited the strongest positive correlation with Macrophages M1 and the strongest negative correlation with Macrophages M2. (H-X) Results obtained using the ssGSEA algorithm indicated that CEBPB was positively correlated with most immune cells, with a notable exception being its significant negative correlation with Th17 cells. (Y) Results from the ESTIMATE algorithm indicated that CEBPB showed a significant positive correlation with ESTIMATEScore, ImmuneScore, and StromalScore.

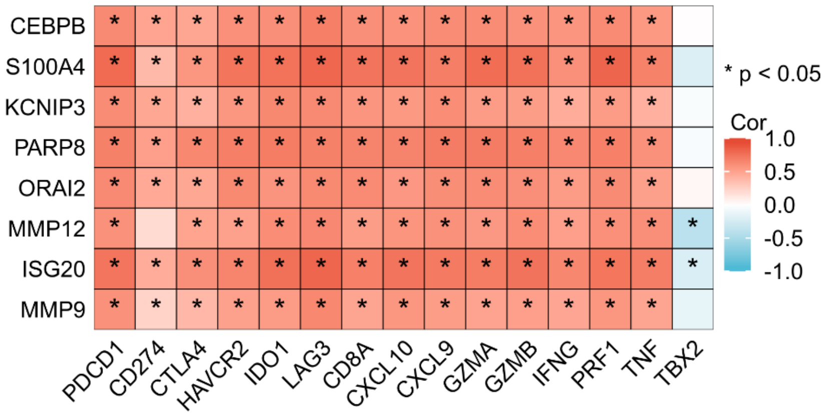

Fig. S10. Correlation heatmap of eight immune-related prognostic genes with immune checkpoint genes and immune activation genes. The results suggested that the prognostic genes were significantly positively correlated with PD-1 (PDCD1), PDL1 (CD274), CTLA4, HAVCR2, IDO1, LAG3, CD8A, CXCL10, CXCL9, GZMA, GZMB, IFNG, PRF1, and TNF. However, there was no significant correlation or a significant negative correlation with TBX2.
